# Supplementary material for: A﻿ TMPRSS2 inhibitor acts as a pan-SARS-CoV-2 prophylactic and therapeutic
Source: Nature. 2022 Mar 28;605(7909):340–8. doi: 10.1038/s41586-022-04661-w (PMC9095466; doi:10.1038/s41586-022-04661-w)
Supplement: Supplementary file 1 — This file includes compound synthesis schemes and characterization of final compounds (Supplementary Schemes 1 to 10); Supplementary Tables that summarize high-resolution mass measurements (Supplementary Tables 1–8); and Supplementary Figures summarizing isotopic profiles, UPLC–MS, and NMR analysis of peptidomimetic compounds (Supplementary Figures 1–27). [file 41586_2022_4661_MOESM1_ESM.pdf]

nature portfolio

https://doi.org/10.1038/s41586-022-04661-w

Supplementary information

A TMPRSS2 inhibitor acts as a pan-SARS-CoV-2 prophylactic and therapeutic

In the format provided by the authors and unedited

Chemical Synthesis of Peptidomimetic Compounds

Abbreviations used: ACN (Acetonitrile), DCM (Dichloromethane), DMP (Dess-Martin periodinane), DIPEA (N,N-Diisopropylethylamine), DMF (N,N-dimethyl formamide), EtOAc (Ethyl acetate), HATU (dimethylamino)-N,N-dimethyl(3H-[1,2,3]triazolo[4,5-b]pyridin-3-yloxy)methaniminium hexafluorophosphate), HCl (Hydrochloric acid), HFIP (Hexafluoroisopropanol), iPrOH (Isopropanol), UPLC-Ms (Ultra perfomance liquid chromatography mass spectrum), min (Minutes), MeOH (Methanol), MsCl (Methanesulfonyl chloride).

Synthesis of N-0130

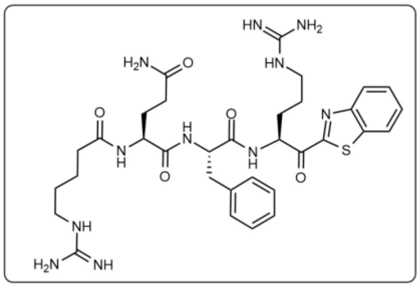

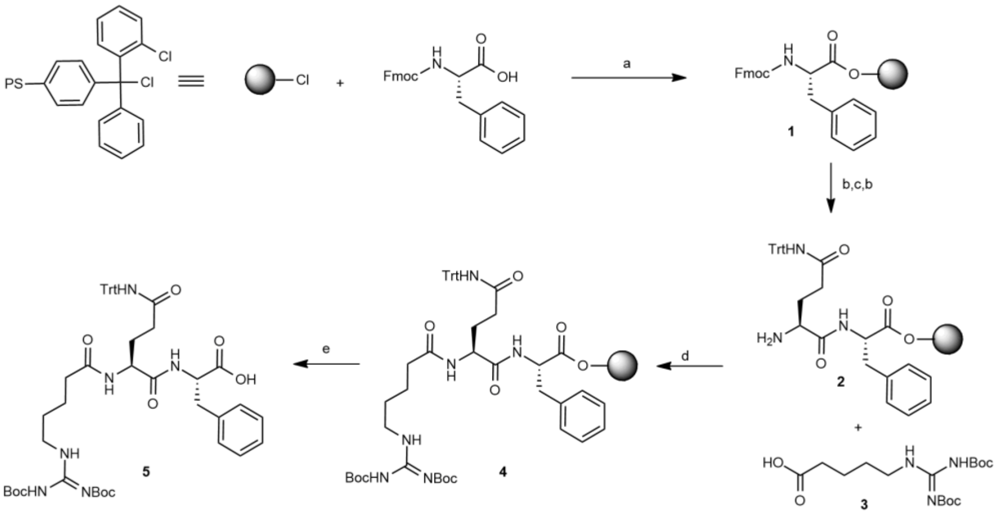

Supplementary Scheme 1. Solid phase synthesis of (H)Arg(Boc)$_2$-Gln(Trt)-Phe (5). Reagents and conditions: (a) DCM, DIPEA (b) Piperidine/DMF (20:80) (c) Fmoc-Gln(Trt)-OH, HATU, DIPEA, DMF (d) **3**, HATU, DIPEA, DMF (e) HFIP/DCM (20:80).

Fmoc-Phe-Resin, Intermediate 1:

To 10 g of CTC Resin with a loading of 1.2 mmol/g were added Fmoc-Phe-OH (9.3 g, 24 mmol, 2 eqs.) dissolved in DCM (approximately 10 mL per gram of resin), and DIPEA (6.3 mL, 36 mmol, 3 eqs.). The mixture was shaken vigorously for 30 - 60 min. To endcap any remaining reactive trityl chloride groups, HPLC grade methanol was added (0.8 mL per gram of resin) and mixed for 15 minutes. The resin was filtered and washed with 3 x DCM, 2 x DMF, 2 x DCM, 3 x iPrOH, 3 x DCM, then dried *in vacuo*.

NH_2-Gln(Trt)Phe-Resin, Intermediate 2:

A solution of DMF/piperidine (20%) was added to the resin, which was then gently shaken for 30 minutes. The resin was filtered and washed with 3 x DMF, iPrOH, 3x DCM then dried *in vacuo.* A solution of Fmoc-Gln(Trt)-OH (14.6 g, 24 mmol, 2 eqs.), HATU (9.3 g, 24 mmol, 2 eqs.) and DIPEA (1.05 mL, 6 mmol, 5 eqs.) in DMF (approximately 10 mL per gram of resin) was added on resin. The mixture was shaken for 2 h, filtered, then washed with 3 x DMF, iPrOH, 3 x DCM then dried *in vacuo.*

(H)Arg$(Boc)_2$-Gln(Trt)-Phe-Resin, Intermediate 4:

A solution of DMF/piperidine (20%) was added to the resin, which was then gently shaken for 30 minutes. The resin was filtered and washed with 3 x DMF, iPrOH, 3x DCM then dried *in vacuo.* A solution of (H)Arg$(Boc)_2$-OH **3** (8.7 g, 24 mmol, 3 eqs.), HATU (9.3 g, 24 mmol, 3 eqs.) and DIPEA (1.05 mL, 6 mmol, 5 eqs.) were dissolved in DMF (approximately 10 mL per gram of resin), was added on resin. The resin was shaken for 2 h, filtered, washed with 3 x DMF, iPrOH, 3 x DCM then dried *in vacuo.*

(H)Arg$(Boc)_2$-Gln(Trt)-Phe-OH Intermediate 5:

To 10 g of derivatized resin was added a solution 20% HFIP in DCM and shaken for 45 minutes. After removal of the solution, the resin was washed with DCM/HFIP (20%), 3 x DCM. After suspension and co-evaporation in diethylether, the white solid was filtrated and dried in vacuo to give tripeptide **5** as a white solid (8.8 g). The compound is used as it in the next step without purification.

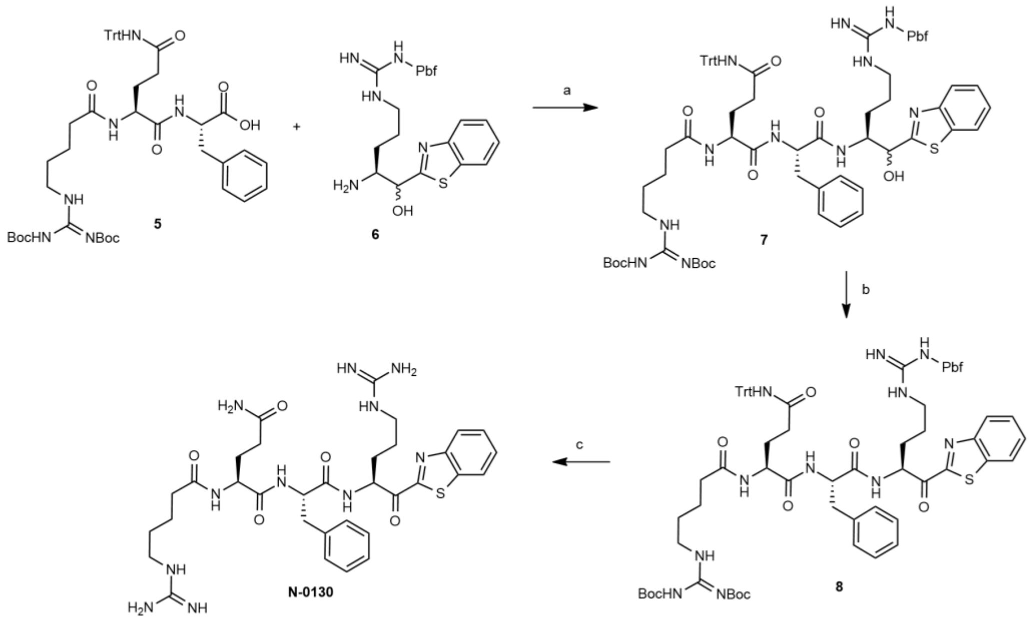

Supplementary Scheme 2. Solution synthesis of N-0130. Reagents and conditions: (a) HATU, DIPEA, DMF, 74% (b) DMP, DCM, 86%. (c) TFA/H_2O (95:5).

Warhead synthesis: Compound **6** was prepared as described in *Duchêne et al.*, J. Med. Chem, 2014, 57 (23), 10198-10204 with minor modifications.

To a solution of intermediate **5** (3.72 g, 4.25 mmol, 1 eq.) in anhydrous DMF were added HATU (1.61 g, 4.25 mmol, 1.1 eq.), NH_2-Arg(Pbf)-C(OH)Bt **6** (2.55 g, 4.68 mmol, 1.1 eq.), and DIPEA (2.2 mL, 12.7 mmol, 3 eqs.) at 0 °C. The mixture was stirred 15 minutes. The protected tetrapeptide was precipitated in cold water (0 °C), filtrated, washed with cold water twice. The filtrate was dissolved in ethyl acetate, washed with citric acid (10%) and brine. The organic phase was dried with sodium sulfate, filtrated and evaporated. The white solid was triturated in ether and purified by flash chromatography (MeOH/DCM 1:99 to MeOH/DCM 5:95). Intermediate **7** is obtained as a white solid (3.2 g, 52%).

DMP (1.2 g, 2.8 mmol, 1.4 eq.) was added to a solution of protected tetrapeptide **7** (2.8 g, 2 mmol, 1 eq.) in DCM for 15 minutes. The solution is washed with water, citric acid 10% and brine. The organic phase is dried with sodium sulfate and evaporated. The residue was triturated in cold ether

and purified by flash chromatography (MeOH/DCM 1:99 to MeOH/DCM 5:95) to give the desired intermediate **8** as a white solid (2.4g, 86%).

2.4 g of intermediate **8** is dissolved in a mixture of 20mL of TFA/H_2O (95:5) and stirred for 1 hour, until completion of the reaction by UPLC-MS. The TFA/H_2O solution is added dropwise to 2 x 35 ml of cold water (0 °C) in two centrifugation tubes and then centrifuged at 4000 rpm for 30 minutes. The supernatant is removed and the white precipitate is dissolved in water, washed with ether and lyophilized. A >95:5 mixture of diastereomer in favor of the S diastereomer of the arginine alpha carbon is obtained (1.3 g).

Compound is purified by reverse phase prep-HPLC MS (C18) using a ACN/water gradient (0.1% TFA) from 10 to 30% of ACN. 27 mg of pure compound is obtained from 50 mg of crude. UPLC-MS retention time: 0.93 min. Purity: >95%

(H)RQFR-Kbt (N-0130):
^{1}H-NMR (400 MHz, d_6-DMSO): δ 8.84 p.p.m. (d, J = 5.6 Hz, 1H), 8.77 (d, J = 7.5 Hz, 1H), 8.68 (s, 1H), 8.58 (d, J = 8.4 Hz, 1H), 8.44 (br. s., 1H), 8.32-8.23 (m, 2H), 7.72-7.60 (m, 2H), 7.40 (br. s., 4H), 7.31 (d, J = 7.3 Hz, 4H), 7.26-7.08 (m, 4H), 6.68 (s, 1H), 6.58 (m, 1H), 5.50-5.41 (m, 1H), 4.33-4.22 (m, 1H), 4.18-4.08 (m, 1H), 3.16-2.95 (m, 6H), 2.24-2.13 (m, 2H), 2.07-1.94 (m, 2H), 1.93-1.83 (m, 2H), 1.83-1.63 (m, 4H), 1.61-1.48 (m, 2H), 1.45-1.32 (m, 2H); ^{13}C-NMR (125 MHz, d_6-DMSO): δ 192.6, 174.0, 172.3, 171.8, 171.7, 164.5, 157.0, 156.9, 153.0, 138.1, 136.4, 129.2, 128.2, 128.0, 127.6, 126.1, 125.3, 123.2, 54.9, 54.4, 53.2, 40.3, 36.7, 35.0, 31.8, 28.0, 27.8, 27.4, 24.7, 22.5; HRMS (*m/z)*: $[M+H]^+$ calcd for $C_{33}H_{45}N_{11}O_5S$, 708.3398; found, 708.3493

Supplementary Table 1. Accurate mass measurement for the compound N-0130.

Compound	N-0130<br>Abundant Ion	N-0130<br>Secondary Ion
Structure	$C_{33}H_{45}N_{11}O_5S$	$C_{33}H_{45}N_{11}O_5S$
Analysis	LC-Qtof	LC-Qtof
Electrospray	ESI +	ESI +
Charge	2; $[M+2H]^{2+}$	1; $[M+H]^{+}$
m/z theorical	354.6735	708.3398
m/z measured	354.6807	708.3493
Δm	0.0072	0.0095
Dissolution solvent	H_2O/MeCN 80/20 +0.1% formic acid (v+v)	

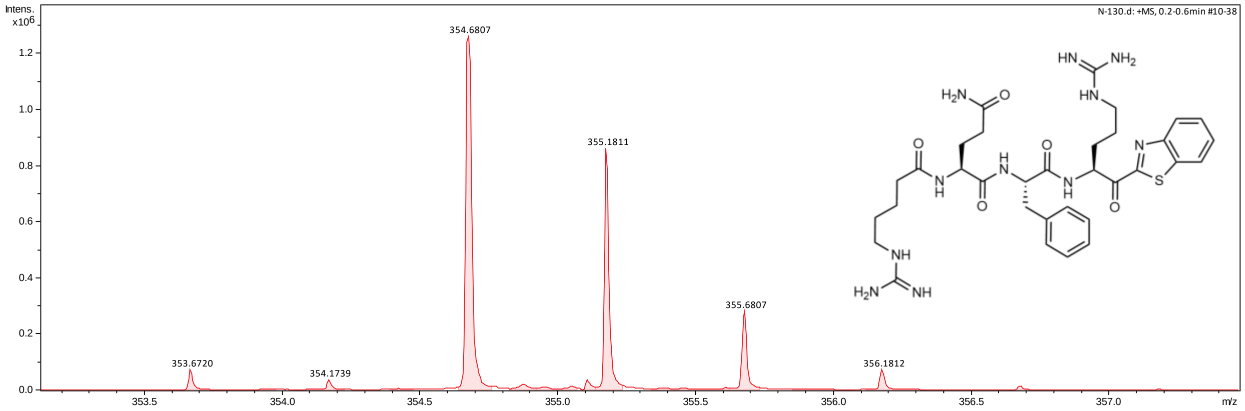

Supplementary Figure 1. Isotopic profile for the most abundant ion (double charged) N-130, $[M+2H]^{2+}$ detected with high-resolution mass spectrometer (Qtof).

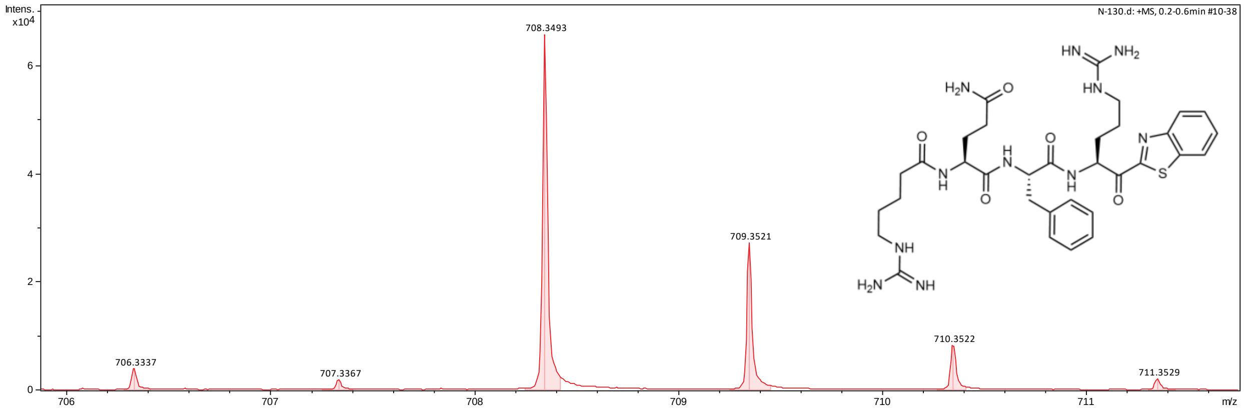

Supplementary Figure 2. Isotopic profile for the secondary ion N-0130, $[M+H]^+$ detected on a high-resolution mass spectrometry (LC-Qtof).

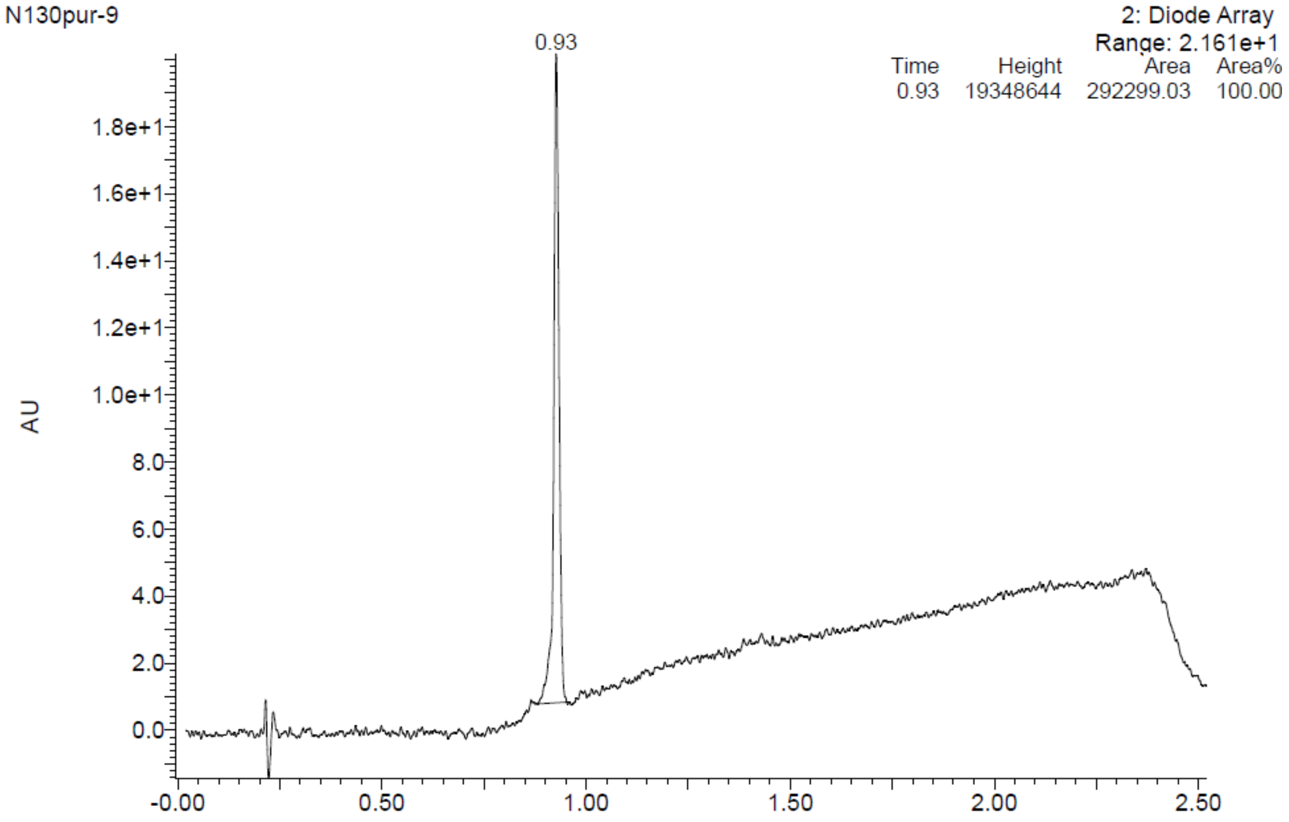

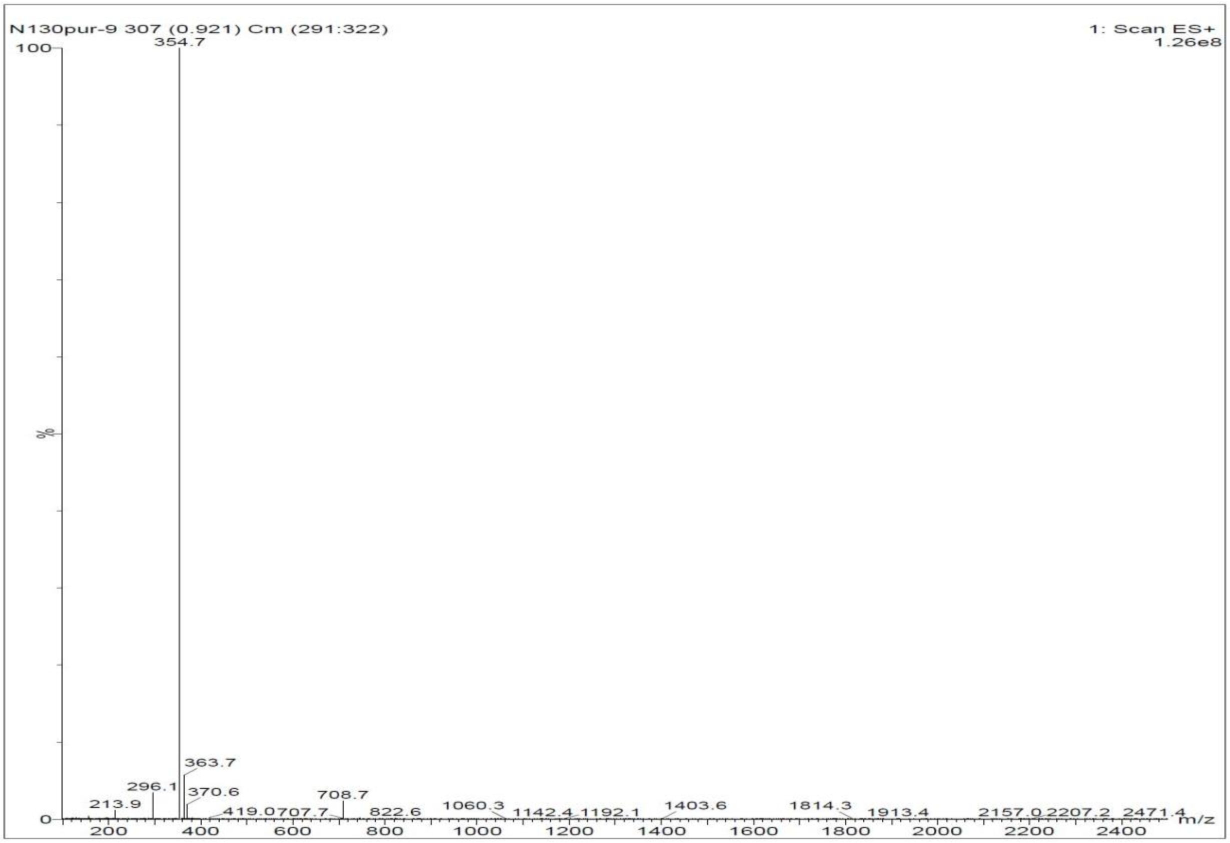

Supplementary Figure 3. UPLC chromatogram and MS of N-0130.

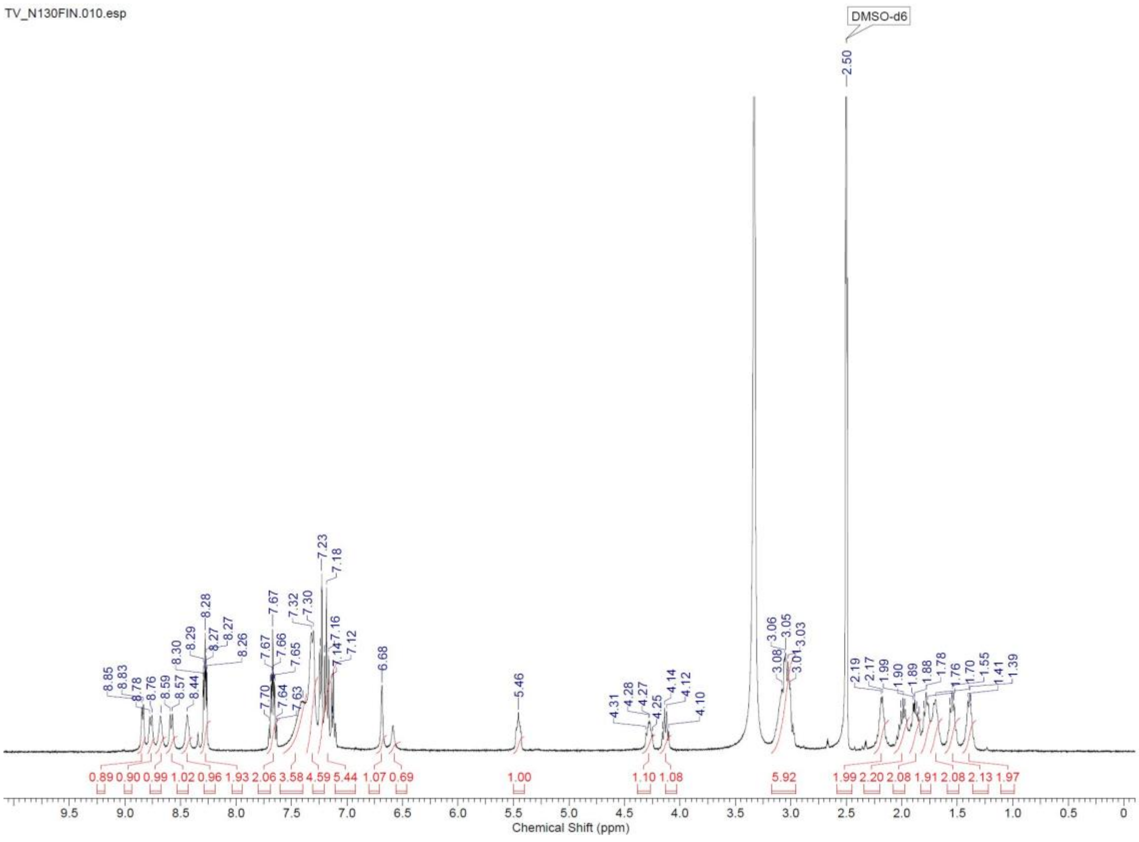

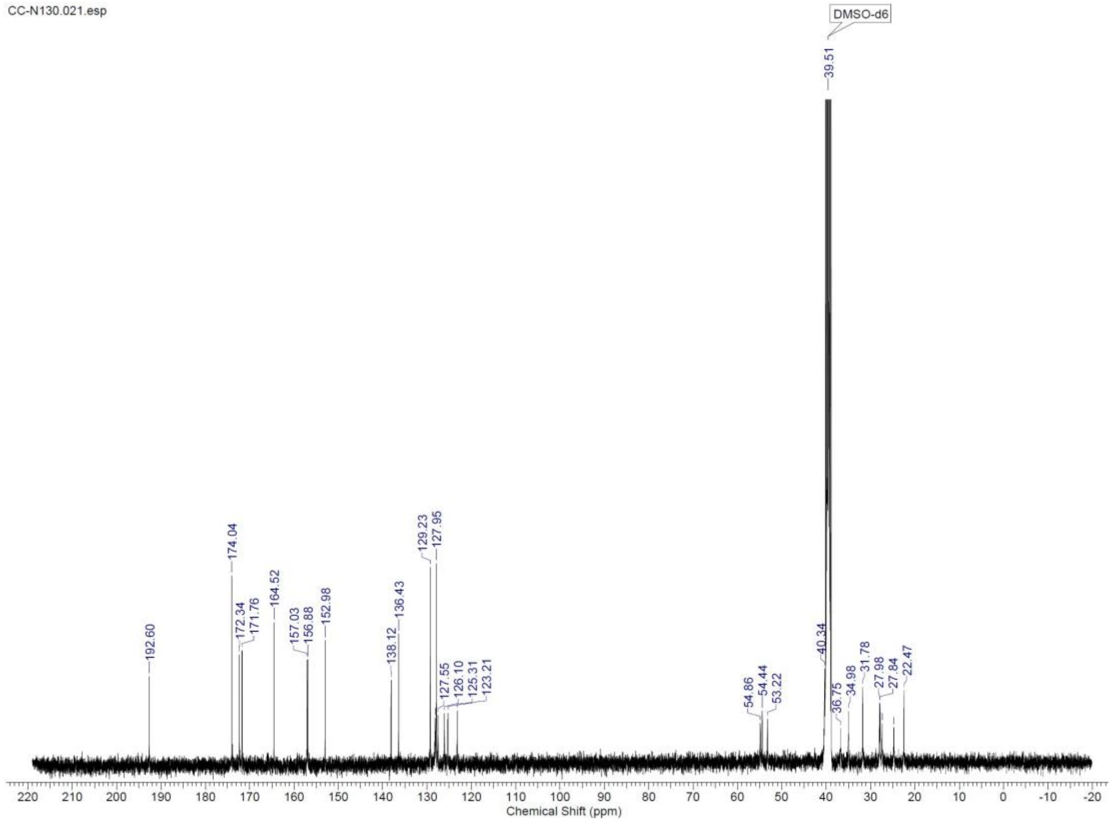

Supplementary Figure 4. ^{1}H-NMR and ^{13}C-NMR of N-0130.

Synthesis of N-0386

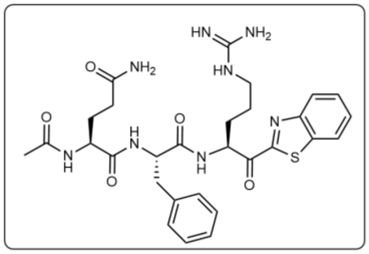

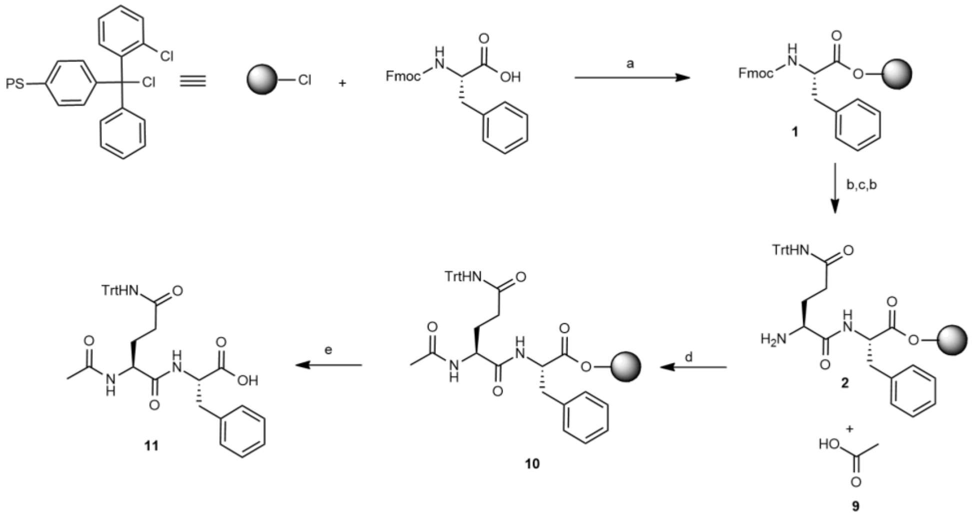

Supplementary Scheme 3. Solid phase synthesis of Ac-Gln(Trt)-Phe (11). Reagents and conditions: (a) DCM, DIPEA (b) Piperidine/DMF (20:80) (c) Fmoc-Gln(Trt)-OH, HATU, DIPEA, DMF (d) **9**, HATU, DIPEA, DMF (e) HFIP/DCM (20:80).

Fmoc-Phe-Resin, Intermediate 1:

To 10 g of CTC Resin with a loading of 1.2 mmol/g were added Fmoc-Phe-OH (9.3 g, 24 mmol, 2 eqs.) dissolved in DCM (approximately 10 mL per gram of resin), and DIPEA (6.3 mL, 36 mmol, 3 eqs.) The mixture was shaken vigorously for 30 - 60 min. To endcap any remaining reactive trityl chloride groups, HPLC grade methanol was added (0.8 mL per gram of resin) and mixed for 15 minutes. The resin was filtered and washed with 3 x DCM, 2 x DMF, 2 x DCM, 3 x iPrOH, 3 x DCM, then dried *in vacuo*.

NH_2-Gln(Trt)Phe-Resin, Intermediate 2:

A solution of DMF/piperidine (20%) was added to the resin, which was then gently shaken for 30 minutes. The resin was filtered and washed with 3 x DMF, iPrOH, 3x DCM then dried *in vacuo.* A solution of Fmoc-Gln(Trt)-OH (14.6 g, 24 mmol, 2 eqs.), HATU (9.3 g, 24 mmol, 2eqs.) and DIPEA (1.05 mL, 6 mmol, 5 eqs.) were dissolved in DMF (approximately 10 mL per gram of resin), was added on resin. The resin was shaken for 2 h, filtered, washed with 3 x DMF, iPrOH, 3 x DCM then dried *in vacuo.*

Ac-Gln(Trt)-Phe-Resin, Intermediate 10:

A solution of DMF/piperidine (20%) was added to the resin, which was then gently shaken for 30 minutes. The resin was filtered and washed with 3 x DMF, iPrOH, 3x DCM then dried *in vacuo.* A solution of Acetic acid **9** (1.8 mL, 30 mmol, 2.5 eqs.), HATU (12 g, 30 mmol, 2.5 eqs.) and DIPEA (10 mL, 60 mmol, 5 eqs.) were dissolved in DMF (approximately 10 mL per gram of resin), was added on resin. The resin was shaken for 2 h, filtered, washed with 3 x DMF, iPrOH, 3 x DCM then dried *in vacuo.*

Ac-Gln(Trt)-Phe-OH Intermediate 11:

To 10 g of derivatized resin was added a solution 20% HFIP in DCM and shaken for 45 minutes. After removal of the solution, the resin was washed with DCM/HFIP (20%), 3 x DCM. After suspension and co-evaporation in diethyl ether, the white solid was filtrated and dried in vacuo to give tripeptide **11** as a white solid (6.7 g). The compound is used as it in the next step without purification.

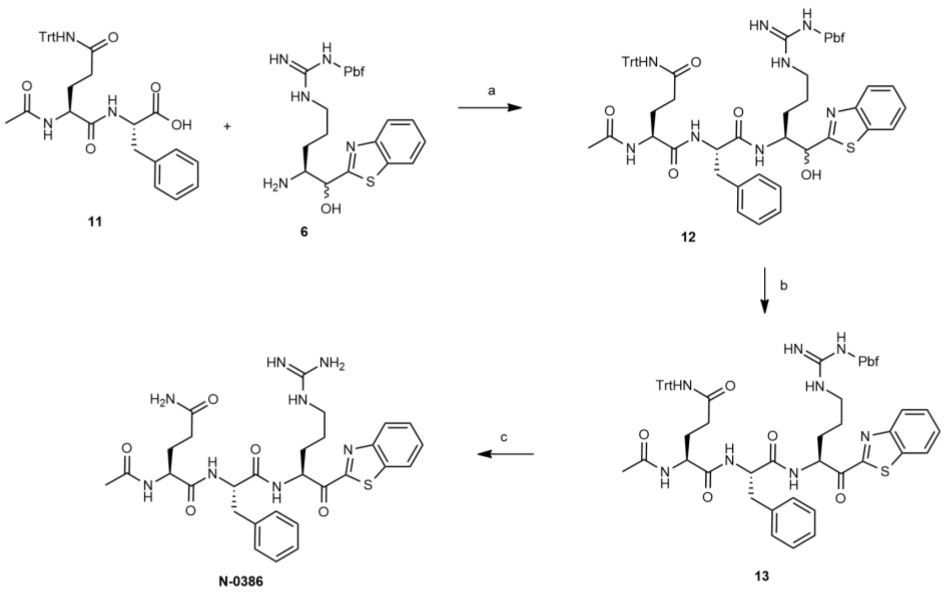

Supplementary Scheme 4. Solution synthesis of N-0386. Reagents and conditions: (a) HATU, DIPEA, DMF, 74% (b) DMP, DCM, 48%. (c) TFA/H_2O (95:5).

To a solution of Intermediate **11** (3.5 g, 6.1 mmol, 1 eq.) in anhydrous DMF (45mL) were added HATU (2.6 g, 6.9 mmol, 1.1 eq.), NH_2-Arg(Pbf)-C(OH)Bt **6** (3.7 g, 6.9 mmol, 1.1 eq.), and DIPEA (3.25 mL, 10.2 mmol, 3 eq.) at 0 °C. The mixture was stirred 15 minutes. The solution was poured in cold water (0 °C), filtrated, washed with cold water twice. The filtrate was dissolved in ether and DCM, washed with citric acid (10%) and brine. The organic phase was dried with sodium sulfate, filtrated and evaporated. The white solid was triturated in ether/hexane. The white solid was filtrated and used as it without purification. (5.3 g)

DMP (3 g, 7 mmol, 1.5 eq.) was added to a solution of protected tetrapeptide **12** (5.2 g, 4.7 mmol, 1 eq.) in DCM for 15 minutes. The solution is washed with water, citric acid 10% and brine. The organic phase is dried with sodium sulfate and evaporated. The residue was triturated in cold ether and purified by flash chromatography (EtOAc/Hexane 10:90 0:100) to give the desired intermediate **13** as a white solid (3.5 g)

3 g of intermediate **13** is dissolved in a mixture of 20 mL of TFA/H_2O (95:5) and stirred for 1 hour, until completion of the reaction by UPLC-MS. The TFA/H_2O solution is added dropwise to 2 x 35

ml of cold water (0 °C) in two centrifugation tubes and then centrifuged at 4000 rpm for 30 minutes. The supernatant is removed and the white precipitate is dissolved in water, washed with ether. A 90:10 mixture of diastereomer in favor of the S diastereomer of the arginine alpha carbon is obtained (2.0 g)

Compound is purified by reverse phase prep-HPLC MS (C18) using a ACN/water gradient (0.1% TFA) from 20 to 40% of ACN. 38 mg of pure compound is obtained from 50 mg of crude. UPLC-MS retention time: 1.20 min. Purity: >95%

Ac-QFR-Kbt (N-0386):

^{1}H-NMR (400 MHz, *d*$_6$-DMSO): δ 8.73 p.p.m. (d, *J* = 6.3 Hz, 1H), 8.40 (s, 1H), 8.31-8.18 (m, 3H), 8.11 (t, *J* = 6.9 Hz, 1H), 8.06 (d, *J* = 7.9 Hz, 1H), 7.72-7.62 (m, 2H), 7.40 (br. s, 3H), 7.30 (s, 1H), 7.26-7.10 (m, 5H), 6.81-6.70 (m, 1H), 5.52-5.35 (m, 1H), 4.62-4.49 (m, 1H), 4.18-4.07 (m, 1H), 3.18-2.74 (m, 3H), 2.10-1.88 (m, 3H), 1.86-1.72 (m, 4H), 1.70-1.46 (m, 3H); ^{13}C-NMR (125 MHz, *d*$_6$-DMSO): δ 193.1, 174.1, 174.0, 171.6, 171.5, 171.4, 169.9, 169.8, 167.2, 164.5, 164.5, 157.1. 153.0, 137.7, 137.5, 136.5, 129.2, 128.4, 128.2, 128.1, 127.7, 126.4, 125.4, 123.3, 54.5, 54.0, 53.6, 52.6, 52.5, 37.8, 37.3, 31.6, 27.8, 27.8, 27.7, 25.0, 22.6; HRMS (*m/z)*: $[M+H]^+$ calcd for $C_{29}H_{36}N_8O_5S$, 609.2602; found, 609.2688

Supplementary Table 2. Accurate mass measurement for the compound N-0386

Compound	N-0386
Structure	$C_{29}H_{36}N_8O_5S$
Analysis	LC-Qtof
Electrospray	ESI +
Charge	1; $[M+H]^+$
m/z theorical	609.2602
m/z measured	609.2688
Δm	0.0086
Dissolution solvent	H_2O/MeCN 80/20 +0.1% formic acid (v+v)

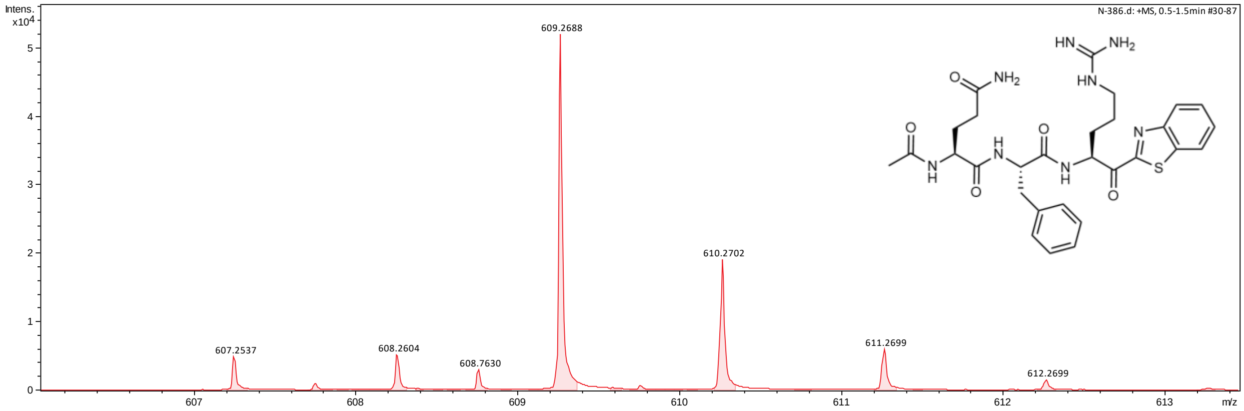

Supplementary Figure 5. Isotopic profile for the compound N-0386, $[M+H]^+$ detected with high-resolution mass spectrometry (Qtof).

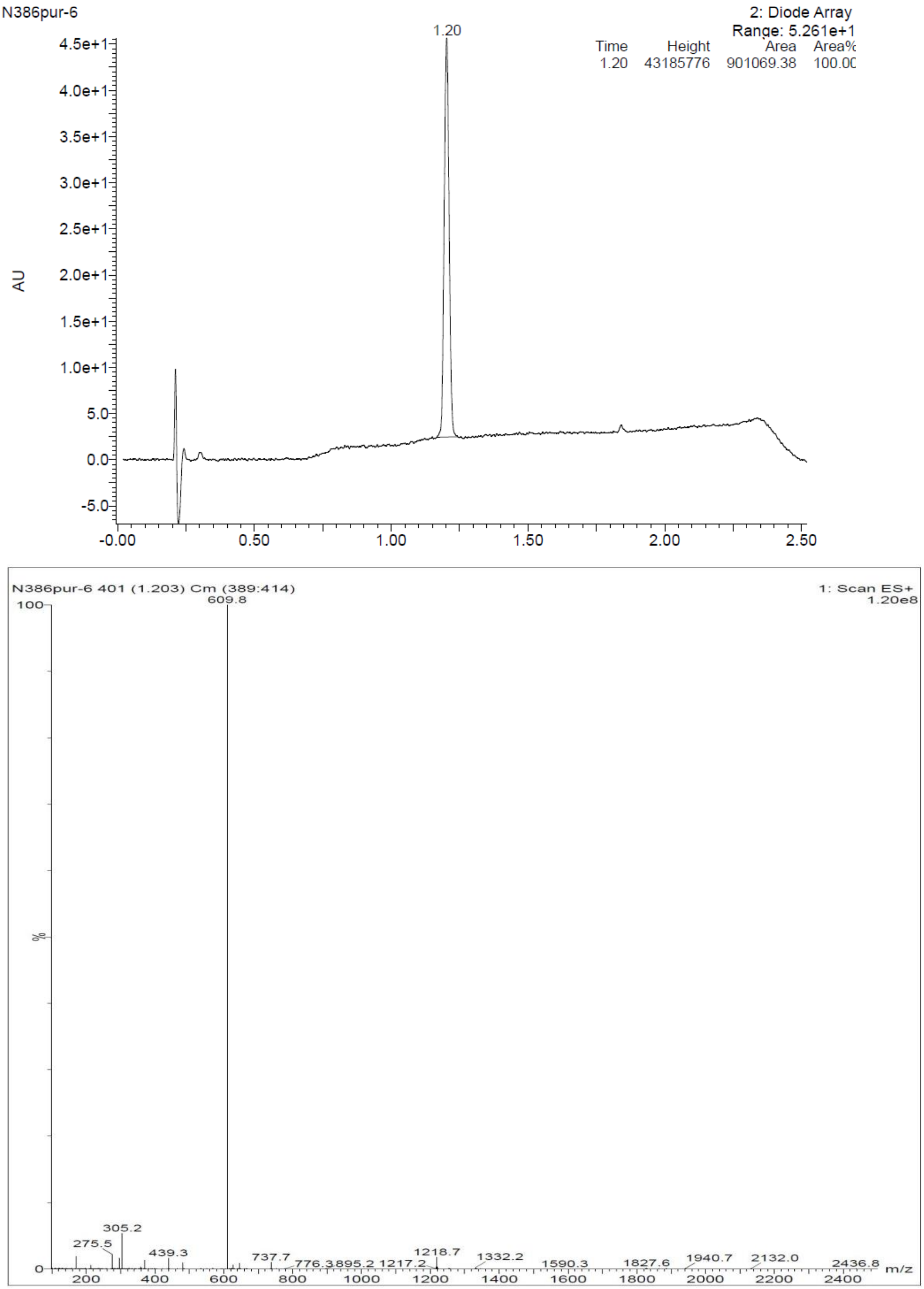

Supplementary Figure 6. UPLC chromatogram and MS of N-0386.

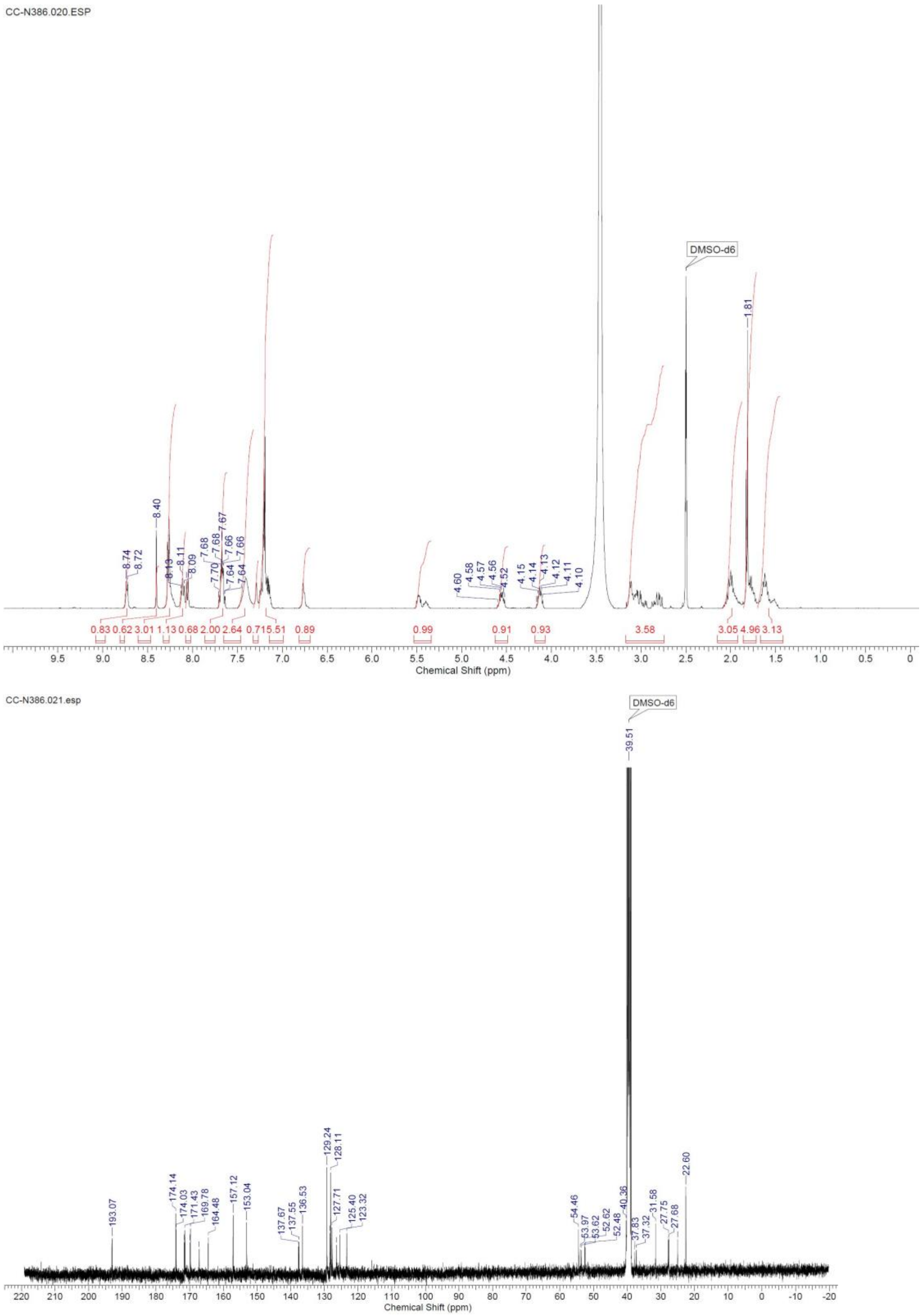

Supplementary Figure 7. ^{1}H-NMR and ^{13}C-NMR of N-0386.

Synthesis of N-0385

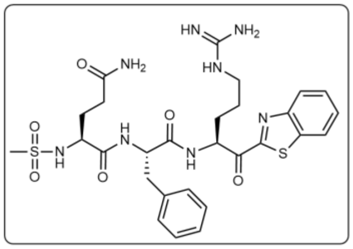

Ms-Q(Trt)-OH 14:

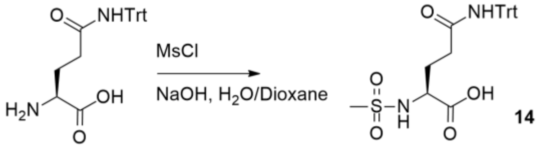

Supplementary Scheme 5. Mesylation of Gln (Trt).

Gln(Trt)-OH (10 g, 25.7 mmol, 1 eq.) is dissolved in 1.5 N NaOH (25 mL) and dioxane (75 mL) was added and cooled at 0 °C. MsCl (2.7 ml, 25.7 mmol, 1 eq.), and NaOH 1.5 N was added dropwise to maintain the pH to 9-10 for 2 h and at room temp. for 2 h. Dioxane is evaporated and ether is added. The precipitate is filtrated and the solid dissolved in DCM/Ether and dried with sodium sulfate. The white solid **14** is obtained (7.2 g) and used in next step without purification

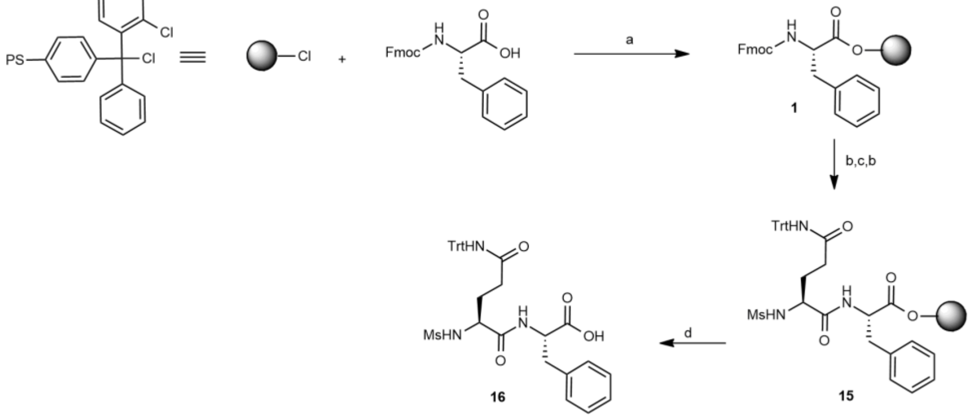

Supplementary Scheme 6. Solid phase synthesis of Ms-Gln(Trt)-Phe (16). Reagents and conditions: (a) DCM, DIPEA (b) Piperidine/DMF (20:80) (c) Ms-Gln(Trt)-OH **14**, HATU, DIPEA, DMF (d) HFIP/DCM (20:80)

Fmoc-Phe-Resin, Intermediate 1:

To 4 g of CTC Resin with a loading of 1.2 mmol/g were added Fmoc-Phe-OH (4.6 g, 12 mmol, 2 eqs.) dissolved in DCM (approximately 10 mL per gram of resin), and DIPEA (3.2 mL, 3 eqs.) The mixture was shaken vigorously for 30 - 60 min. To endcap any remaining reactive trityl chloride groups, HPLC grade methanol was added (0.8 mL per gram of resin) and mixed for 15 minutes. The resin was filtered and washed with 3 x DCM, 2 x DMF, 2 x DCM, 3 x iPrOH, 3 x DCM, then dried *in vacuo*.

Ms-Gln(Trt)Phe-Resin, Intermediate 15:

A solution of DMF/piperidine (20%) was added to the resin, which was then gently shaken for 30 minutes. The resin was filtered and washed with 3 x DMF, iPrOH, 3x DCM then dried *in vacuo.* A solution of Ms-Gln(Trt)-OH **14** (5.35 g, 12 mmol, 3 eqs.), HATU (4.6 g, 12 mmol, 3 eqs.) and DIPEA (3.5 mL, 20 mmol, 5 eqs.) were dissolved in DMF (approximately 10 mL per gram of resin), was added on resin. The resin was shaken for 2 h, filtered, then with 3 x DMF, iPrOH, 3 x DCM then dried *in vacuo.*

Ms-Gln(Trt)-Phe-OH Intermediate 16:

To 4 g of derivatized resin was added a solution 20% HFIP in DCM and shaken for 45 minutes. After removal of the solution, the resin was washed with DCM/HFIP (20%), 3 x DCM. After suspension and co-evaporation in diethyl ether, a white solid was obtained after flash chromatography purification (1.9 g).

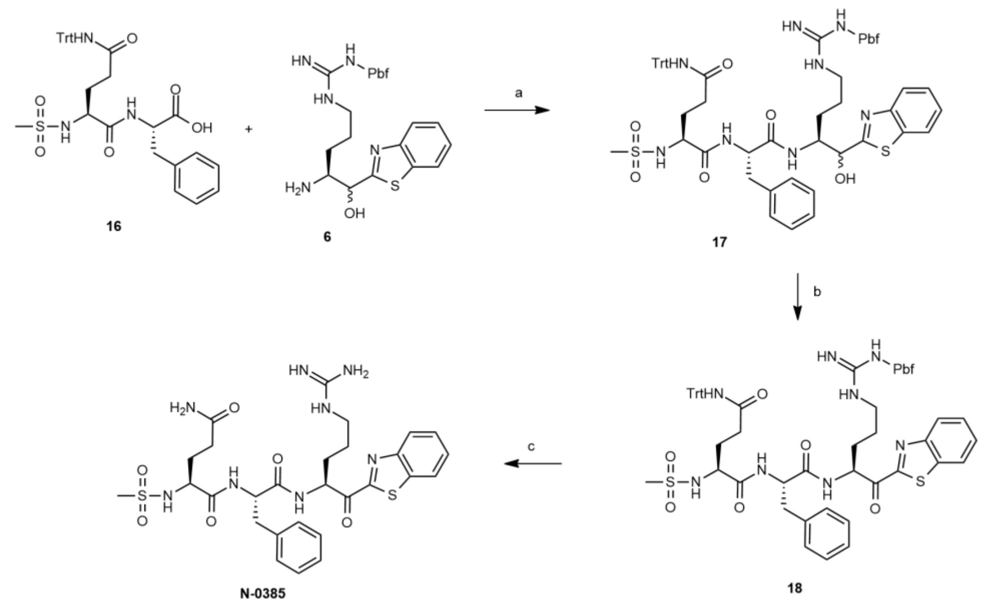

Supplementary Scheme 7. Solution synthesis of N-0385. Reagents and conditions: (a) HATU, DIPEA, DMF, 74% (b) DMP, DCM, 86%. (c) TFA/H_2O (95:5).

To a solution of intermediate **16** (1.8g, 2.9mmol, 1 eq) in anhydrous DMF were added HATU (1.1 g, 2.9 mmol, 1 eq.), NH_2-Arg(Pbf)-C(OH)Bt **6** (1.74 g, 3.2 mmol, 1.1 eq.), and DIPEA (1.5 mL, 8.7 mmol, 3 eqs.) at 0 °C. The mixture was stirred 15 minutes. The solution was poured in cold water (0 °C), filtrated and washed with cold water twice. The filtrate was dissolved in ethyl acetate, washed with citric acid (10%) and brine. The organic phase was dried with sodium sulfate, filtrated and evaporated. The white solid was triturated in ether and filtrated to give intermediate (#?) as a white solid (3.1 g).

DMP (1.6 g, 5.25 mmol, 1.5 eq.) was added to a solution of protected tetrapeptide **17** (2.9 g, 3.5 mmol, 1 eq.) in DCM for 15 minutes. The solution is washed with water, citric acid 10% and brine. The organic phase is dried with sodium sulfate and evaporated. The residue was triturated in cold ether and purified by flash chromatography (EtOAc/Hexane 10:90 0:100) to give the desired intermediate **18** as a white solid (1.6 g)

1.6 g of intermediate **18** is dissolved in a mixture of 20 mL of TFA/H_2O (95:5) and stirred for 1 hour, until completion of the reaction by UPLC-MS. The TFA/H_2O solution is added dropwise to 2 x 35 ml of cold water (0 °C) in two centrifugation tubes and then centrifuged at 4000 rpm for 30

minutes. The supernatant is removed and the white precipitate is dissolved in water, washed with ether and lyophilized. A >95:5 mixture of diastereomer in favor of the S diastereomer of the arginine alpha carbon is obtained (0.8 g).

Compound is purified by reverse phase prep-HPLC MS (C18) using a ACN/water gradient (0.1% TFA) from 20 to 40% of ACN. 32 mg of pure compound is obtained from 50 mg of crude. UPLC-MS retention time: 1.22 min. Purity: >95%

Ms-QFR-Kbt (N-0385):

^{1}H-NMR (400 MHz, d_6-DMSO): δ 9.04 p.p.m. (d, J = 5.8 Hz, 1H), 8.97 - 8.85 (m, 1H), 8.42-8.30 (m, 1H), 8.29 - 8.21 (m, 2H), 7.71-7.57 (m, 3H), 7.37-7.23 (m, 6H), 7.22-7.08 (m, 4H), 6.72 (br. s., 1H), 5.53-5.41 (m, 1H), 4.60-4.52 (m, 1H), 3.74-3.62 (m, 1H), 3.15-2.87 (m, 3H), 2.50 (s, 3H), 2.15-2.04 (m, 1H), 2.04-1.92 (m, 2H), 1.91-1.72 (m, 2H), 1.71-1.56 (m, 3H); ^{13}C-NMR (125 MHz, d_6-DMSO): δ 192.9, 173.9, 171.9, 171.1, 164.5, 156.9, 153.0, 137.9, 136.4, 129.3, 128.3, 128.01, 128.0, 127.6, 126.2, 125.3, 123.3, 56.7, 54.7, 54.5, 40.4, 37.4, 31.6, 28.9, 27.4, 24.9; HRMS (*m/z)*: $[M+H]^+$ calcd for $C_{28}H_{36}N_8O_6S_2$, 645.2271; found, 645.2371

Supplementary Table 3. Accurate mass measurement of the compound N-0385

Compound	N-0385
Structure	$C_{28}H_{36}N_8O_6S_2$
Analysis	LC-Qtof
Electrospray	ESI +
Charge	1; $[M+H]^+$
m/z theorical	645.2271
m/z measured	645.2371
Δm	0.0100
Dissolution solvent	H_2O/MeCN 80/20 +0.1% formic acid (v+v)

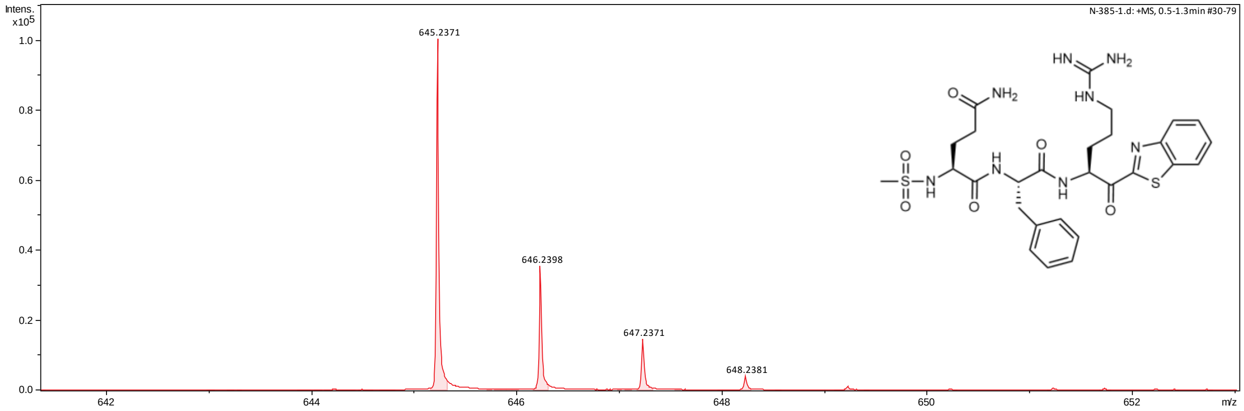

Supplementary Figure 8. Isotopic profile for the compound N-0385, $[M+H]^+$ detected with high-resolution mass spectrometry (Qtof).

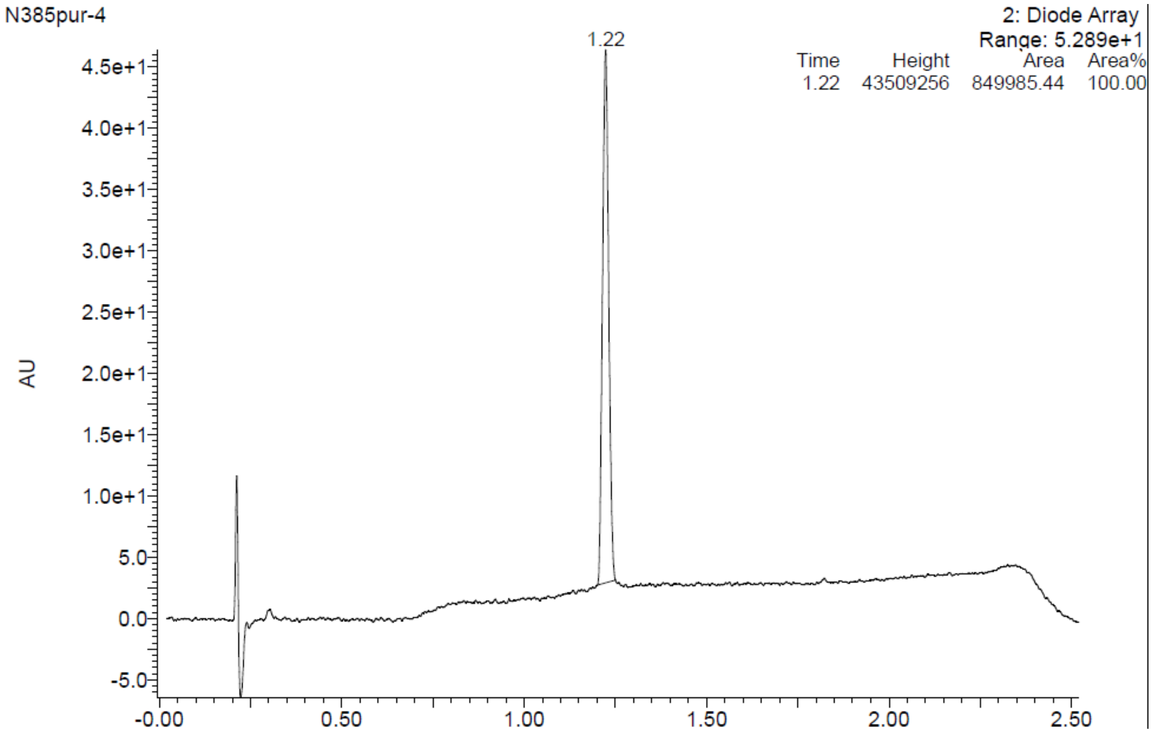

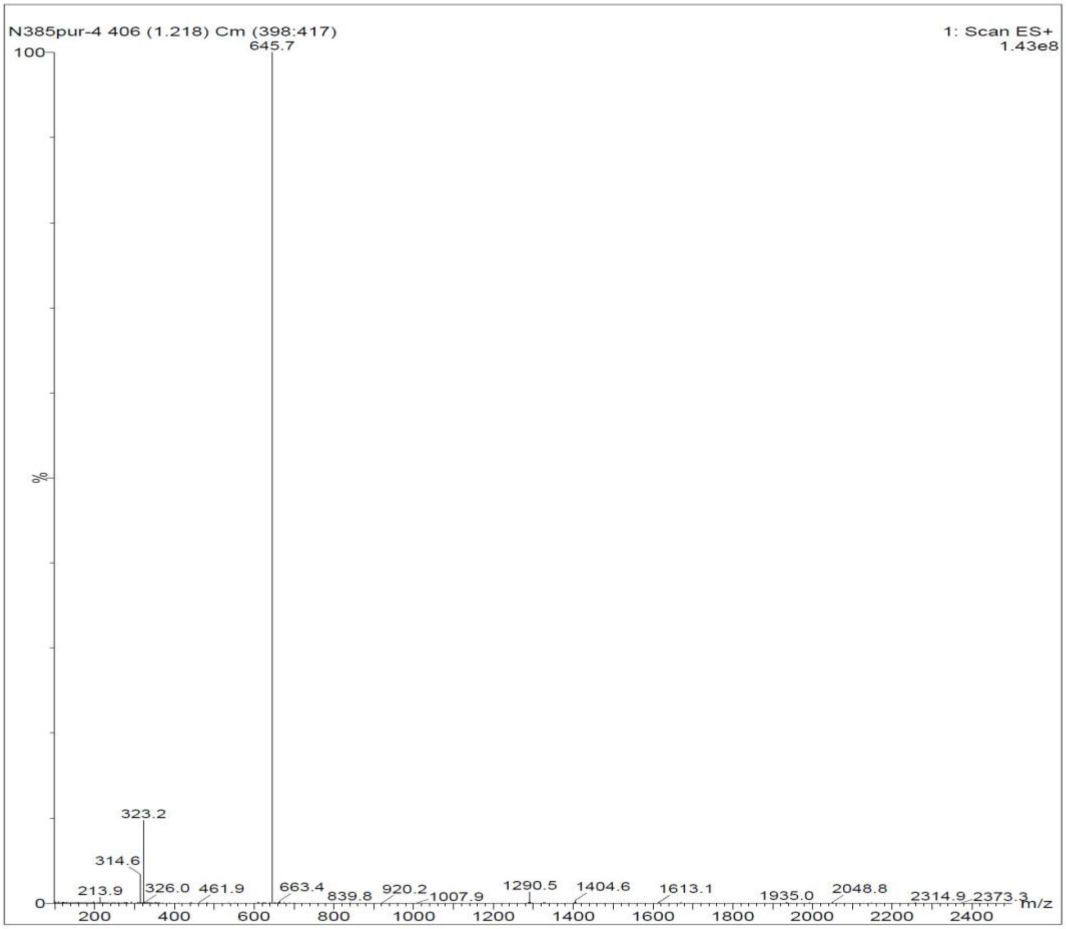

Supplementary Figure 9. UPLC chromatogram and MS of N-0385.

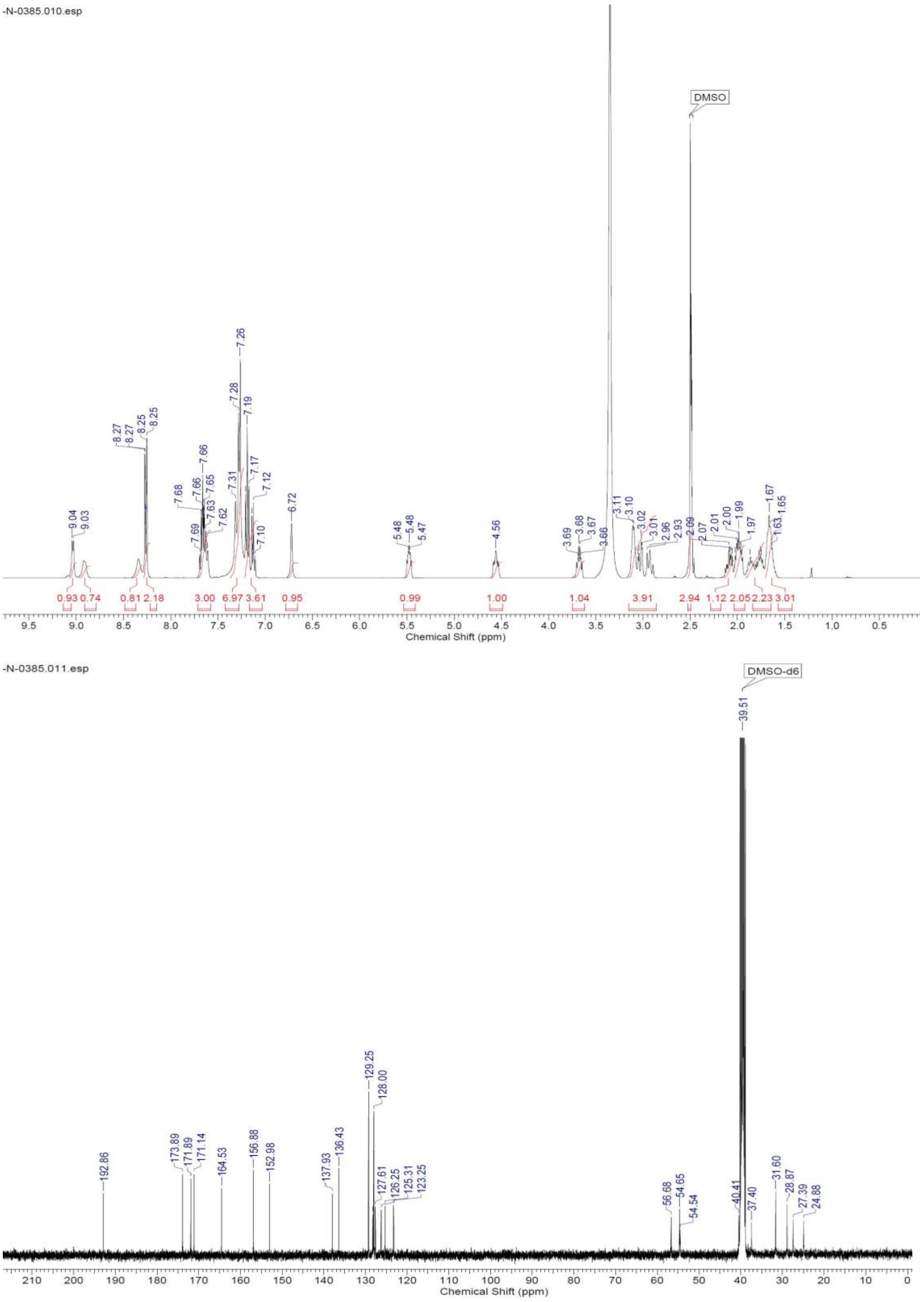

Supplementary Figure 10. ^{1}H-NMR and ^{13}C-NMR of N-0385.

<u>Synthesis of N-0438</u>

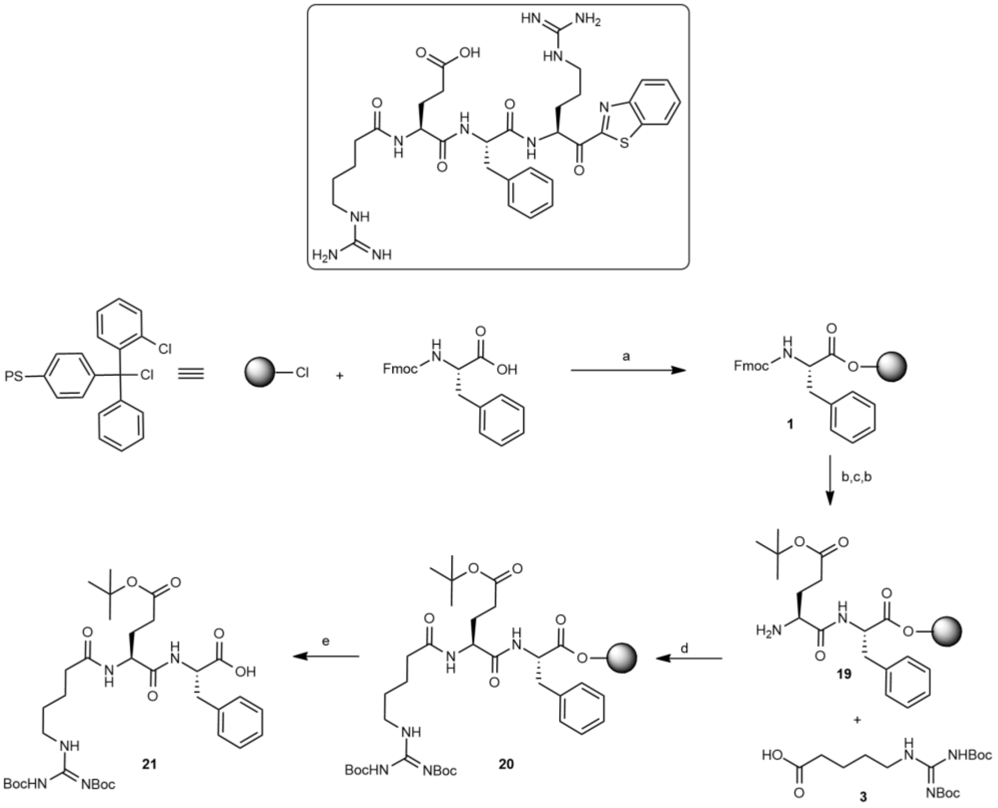

Supplementary Scheme 8. Solid phase synthesis of (H)Arg(Boc)$_2$-Glu(tBu)-Phe (21). Reagents and conditions: (a) DCM, DIPEA (b) Piperidine/DMF (20:80) (c) Fmoc-Glu(tBu)-OH, HATU, DIPEA, DMF (d) **8**, HATU, DIPEA, DMF (e) HFIP/DCM (20:80).

Fmoc-Phe-Resin, Intermediate 1:

To 10 g of CTC Resin with a loading of 1.2 mmol/g were added Fmoc-Phe-OH (9.3 g, 24 mmol, 2 eqs.) dissolved in DCM (approximately 10 mL per gram of resin), and DIPEA (6.3 mL, 36 mmol, 3 eqs.). The mixture was shaken vigorously for 30 - 60 min. To endcap any remaining reactive trityl chloride groups, HPLC grade methanol was added (0.8 mL per gram of resin) and mixed for 15 minutes. The resin was filtered and washed with 3 x DCM, 2 x DMF, 2 x DCM, 3 x iPrOH, 3 x DCM, then dried *in vacuo*.

NH_2-Glu(tBu)Phe-Resin, Intermediate 19:

A solution of DMF/piperidine (20%) was added to the resin, which was then gently shaken for 30 minutes. The resin was filtered and washed with 3 x DMF, iPrOH, 3x DCM then dried *in vacuo.* A solution of Fmoc-Glu(tBu)-OH (16.7 g, 24 mmol, 2 eqs.), HATU (9.3 g, 24 mmol, 2 eqs.) and DIPEA (1.05 mL, 6 mmol, 5 eqs.) were dissolved in DMF (approximately 10 mL per gram of resin), was added on resin. The resin was shaken for 2 h, filtered, washed with 3 x DMF, iPrOH, 3 x DCM then dried *in vacuo.*

(H)Arg$(Boc)_2$-Glu(tBu)-Phe-Resin, Intermediate 20:

A solution of DMF/piperidine (20%) was added to the resin, which was then gently shaken for 30 minutes. The resin was filtered and washed with 3 x DMF, iPrOH, 3x DCM then dried *in vacuo.* A solution of (H)Arg$(Boc)_2$-OH **3** (8.7 g, 24 mmol, 3 eqs.), HATU (9.3 g, 24 mmol, 3 eqs.) and DIPEA (1.05 mL, 6 mmol, 5 eqs.) were dissolved in DMF (approximately 10 mL per gram of resin), was added on resin. The resin was shaken for 2 h, filtered, washed with 3 x DMF, iPrOH, 3 x DCM then dried *in vacuo.*

(H)Arg$(Boc)_2$-Glu(tBu)-Phe-OH Intermediate 21:

To 10 g of derivatized resin was added a solution 20% HFIP in DCM and shaken for 45 minutes. After removal of the solution, the resin was washed with DCM/HFIP (20%), 3 x DCM. After suspension and co-evaporation in diethyl ether, the white solid was filtrated and dried in vacuo to give tripeptide **21** as a white solid (7.9 g). The compound is used as it in the next step without purification.

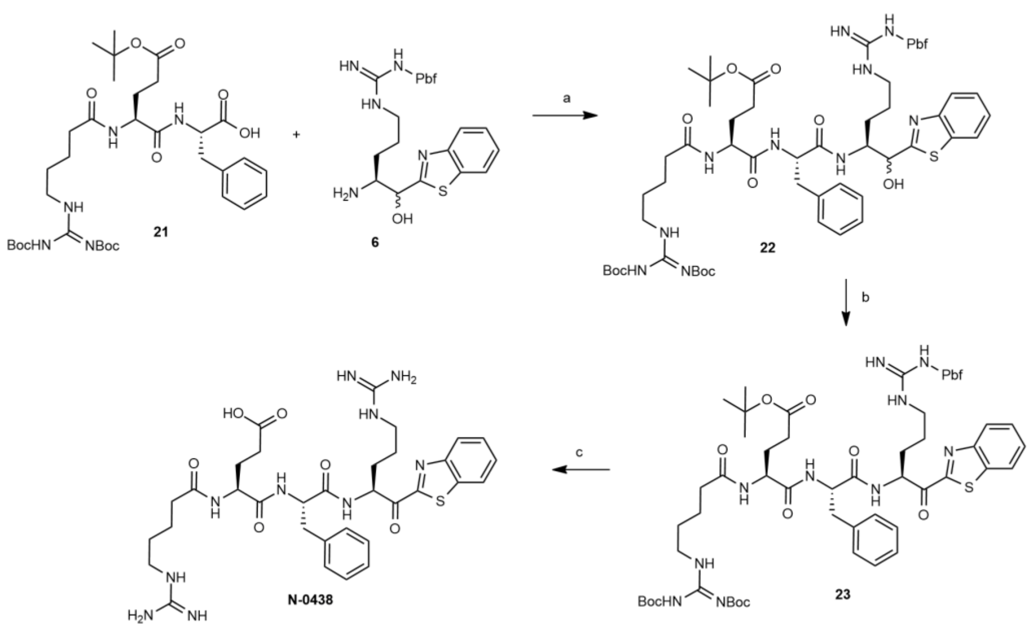

Supplementary Scheme 9. Solution synthesis of N-0438. Reagents and conditions: (a) HATU, DIPEA, DMF, 74% (b) DMP, DCM, 86%. (c) TFA/H_2O (95:5).

To a solution of Intermediate **21** (3.92 g, 4.25 mmol, 1 eq.) in anhydrous DMF were added HATU (1.61 g, 4.25 mmol, 1.1 eq.), NH_2-Arg(Pbf)-C(OH)Bt **6** (2.55 g, 4.68 mmol, 1.1 eq.), and DIPEA (2.2 mL, 12.7 mmol, 3 eqs.) at 0 °C. The mixture was stirred 15 minutes. The protected tetrapeptide was precipitated in cold water (0 °C), filtrated and washed with cold water twice. The filtrate was dissolved in ethyl acetate, washed with citric acid (10%) and brine. The organic phase was dried with sodium sulfate, filtrated and evaporated. The white solid was triturated in ether and purified by flash chromatography (MeOH/DCM 1:99 to MeOH/DCM 5:95). Intermediate **22** is obtained as a white solid (2.9 g, 52%).

DMP (1.04 g, 2.46 mmol, 1.4 eq.) was added to a solution of protected tetrapeptide **22** (2.2 g, 1.64 mmol, 1 eq.) in DCM for 15 minutes. The solution is washed with water, citric acid 10% and brine. The organic phase is dried with sodium sulfate and evaporated. The residue was triturated in cold ether and purified by flash chromatography (MeOH/DCM 1:99 to MeOH/DCM 5:95) to give the desired intermediate **23** as a white solid (1.17 g, 64%).

1.13 g of intermediate **23** is dissolved in a mixture of 10 mL of TFA/H_2O (95:5) and stirred for 1 hour, until completion of the reaction by UPLC-MS. The TFA/H_2O solution is added dropwise to 2 x 35 ml of cold water (0 °C) in two centrifugation tubes and then centrifuged at 4000 rpm for 30 minutes. The supernatant is removed and the white precipitate is dissolved in water, washed with ether and lyophilized. A >95:5 mixture of diastereomer in favor of the S diastereomer of the arginine alpha carbon is obtained.

Compound is purified by reverse phase prep-HPLC MS (C18) using an ACN/water gradient (0.1% TFA) from 10 to 30% of ACN. 311 mg of pure compound is obtained. UPLC-MS retention time: 0.97 min. Purity: >95%

(H)REFR-Kbt (N-0438):

^{1}H-NMR (400 MHz, d_6-DMSO): δ 8.67 p.p.m. (d, *J* = 6.8 Hz, 1H), 8.30-8.24 (m, 2H), 8.01 (d, *J* = 7.7 Hz, 2H), 7.74 (t, *J* = 5.2 Hz, 1H), 7.71-7.63 (m, 3H), 7.53-6.98 (m, 4H), 7.26-7.14 (m, 7H), 5.44 (ddd, *J* = 9.2, 6.9, 4.4 Hz, 1H), 4.62 (td, *J* = 8.5, 5.7 Hz, 1H), 4.21 (td, *J* = 8.0, 5.8 Hz, 1H), 3.13-3.03 (m, 4H), 2.97 (dd, *J* = 13.7, 5.5 Hz, 1H), 2.83 (dd, *J* = 13.6, 8.8 Hz, 1H), 2.21-2.05 (m, 4H), 1.98-1.87 (m, 1H), 1.84-1.73 (m, 1H), 1.72-1.59 (m, 2H), 1.55-1.37 (m, 7H); ^{13}C-NMR (125 MHz, d_6-DMSO): δ 193.0, 174.0, 172.2, 171.1, 164.4, 158.6, 158.3, 156.8, 153.0, 137.4, 136.5, 129.2, 128.3, 128.1, 127.6, 126.3, 125.3, 123.2, 118.6, 115.6, 54.9, 54.2, 53.7, 51.9, 37.9, 34.5, 30.2, 28.0, 27.7, 27.3, 25.0, 22.2; HRMS (*m/z)*: $[M+H]^+$ calcd for $C_{33}H_{44}N_{10}O_6S$, 709.3238; found, 709.3325

Supplementary Table 4. Accurate mass measurement for the compound N-0438.

Compound	N-0438 Abundant ion	N-0438 Secondary Ion
Structure	$C_{33}H_{44}N_{10}O_6S$	$C_{33}H_{44}N_{10}O_6S$
Analyse	LC-Qtof	LC-Qtof
Electrospray	ESI +	ESI +
Charge	2; $[M+2H]^{2+}$	1; $[M+H]^+$
m/z theorical	355.1655	709.3238
m/z measured	355.1718	709.3325
Δm	0.0063	0.0087
Dissolution solvent	H_2O/MeCN 80/20 +0.1% formic acid (v+v)	

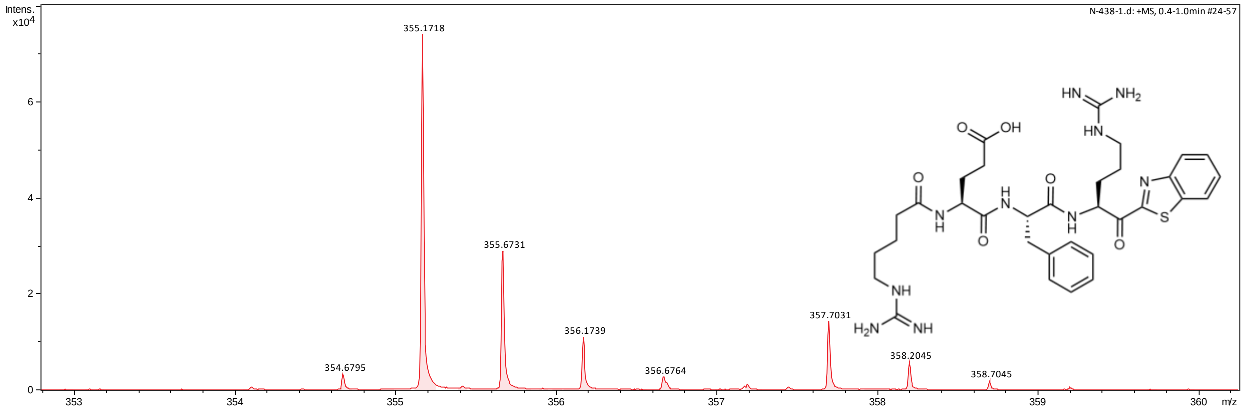

Supplementary Figure 11. Isotopic profile for the most abundant ion (double charged) N-0438, $[M+2H]^{2+}$ detected with high-resolution mass spectrometry (Qtof)

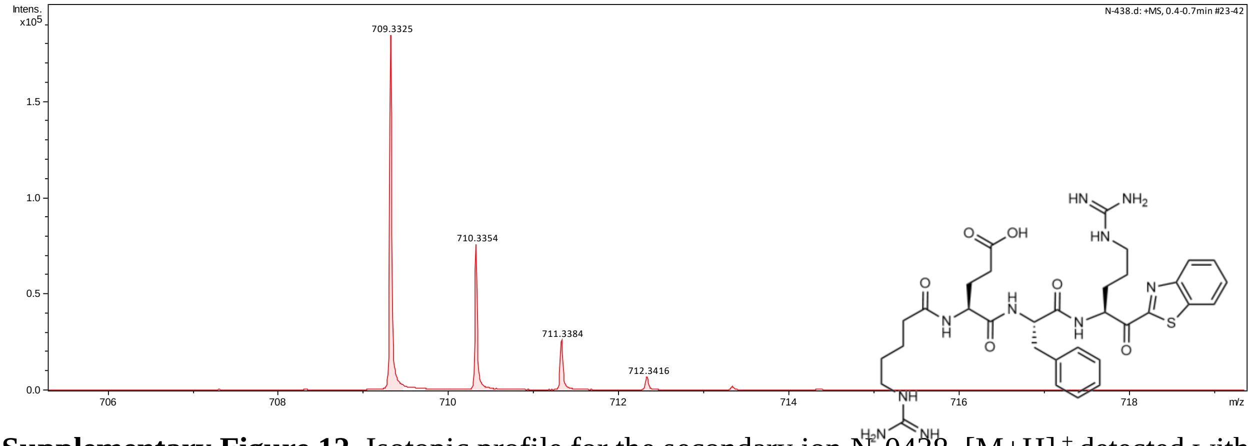

Supplementary Figure 12. Isotopic profile for the secondary ion N-0438, $[M+H]^+$ detected with high-resolution mass spectrometry (Qtof).

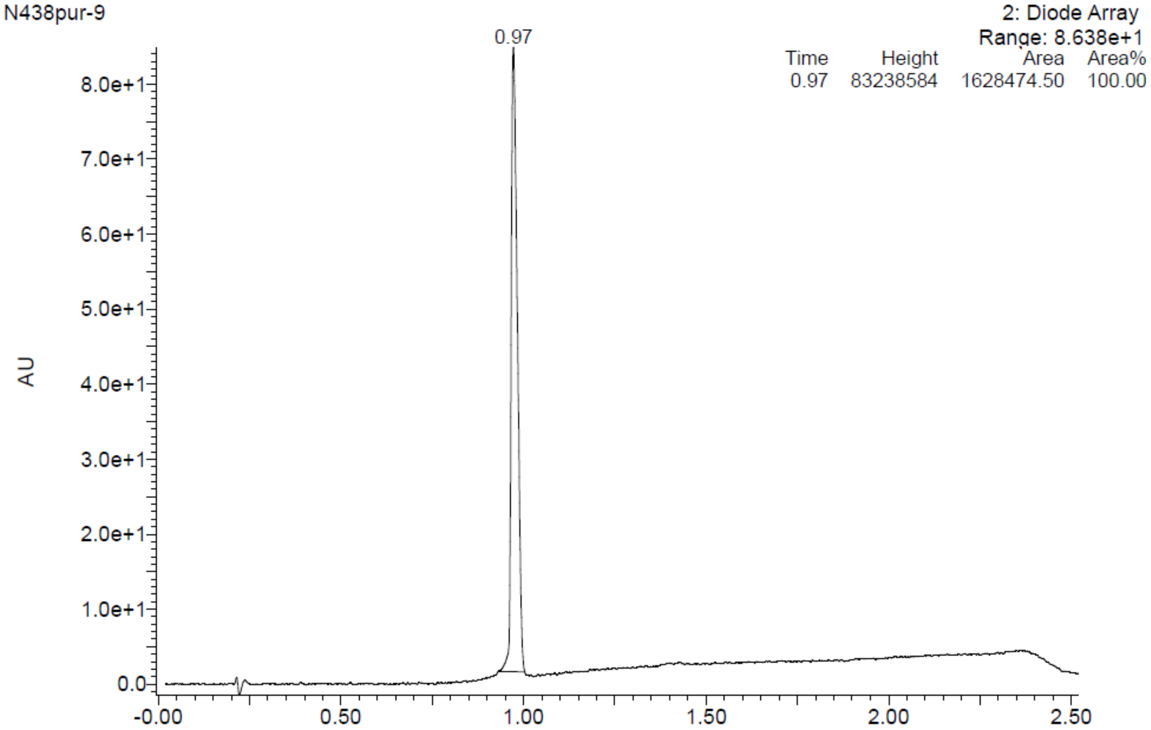

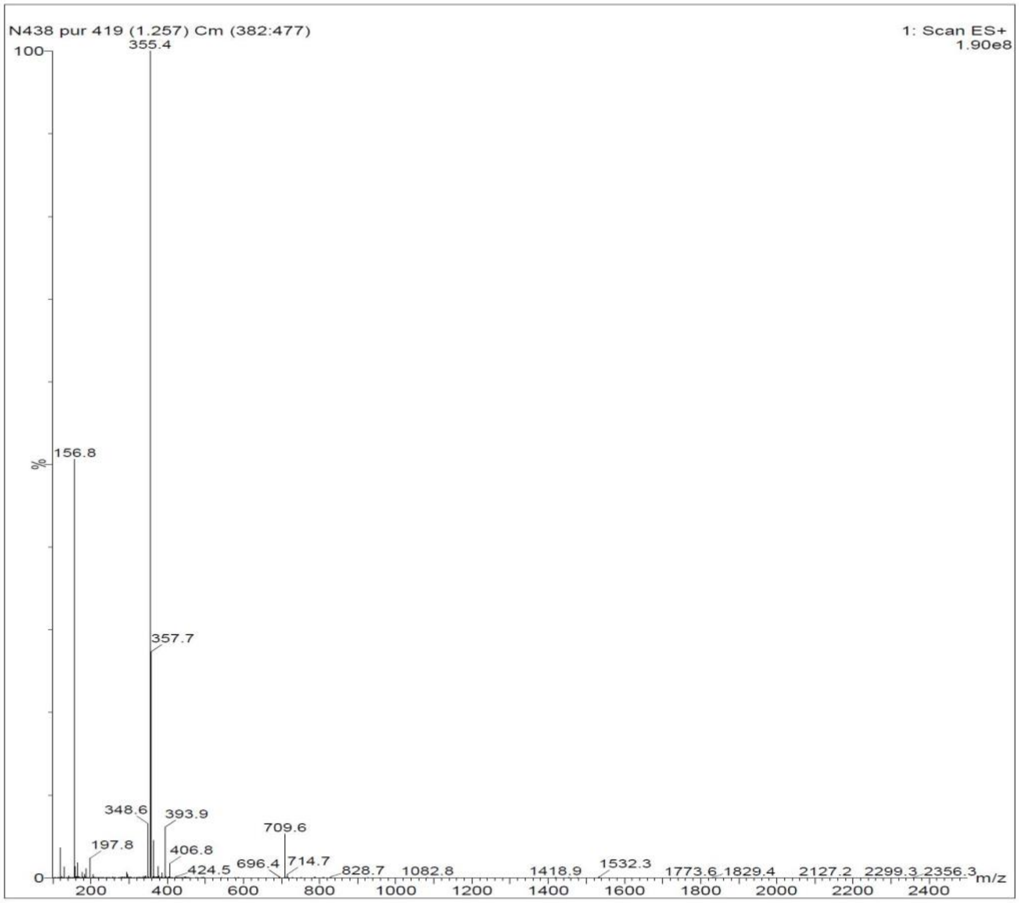

Supplementary Figure 13. UPLC chromatogram and MS of N-0438.

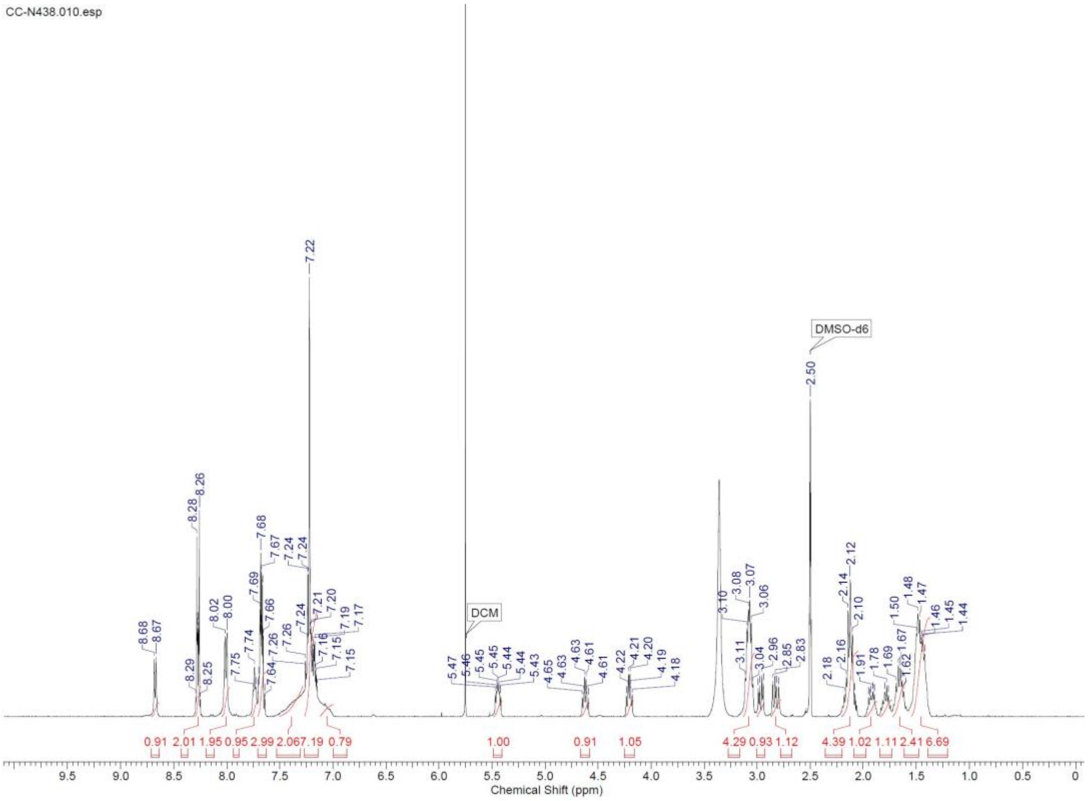

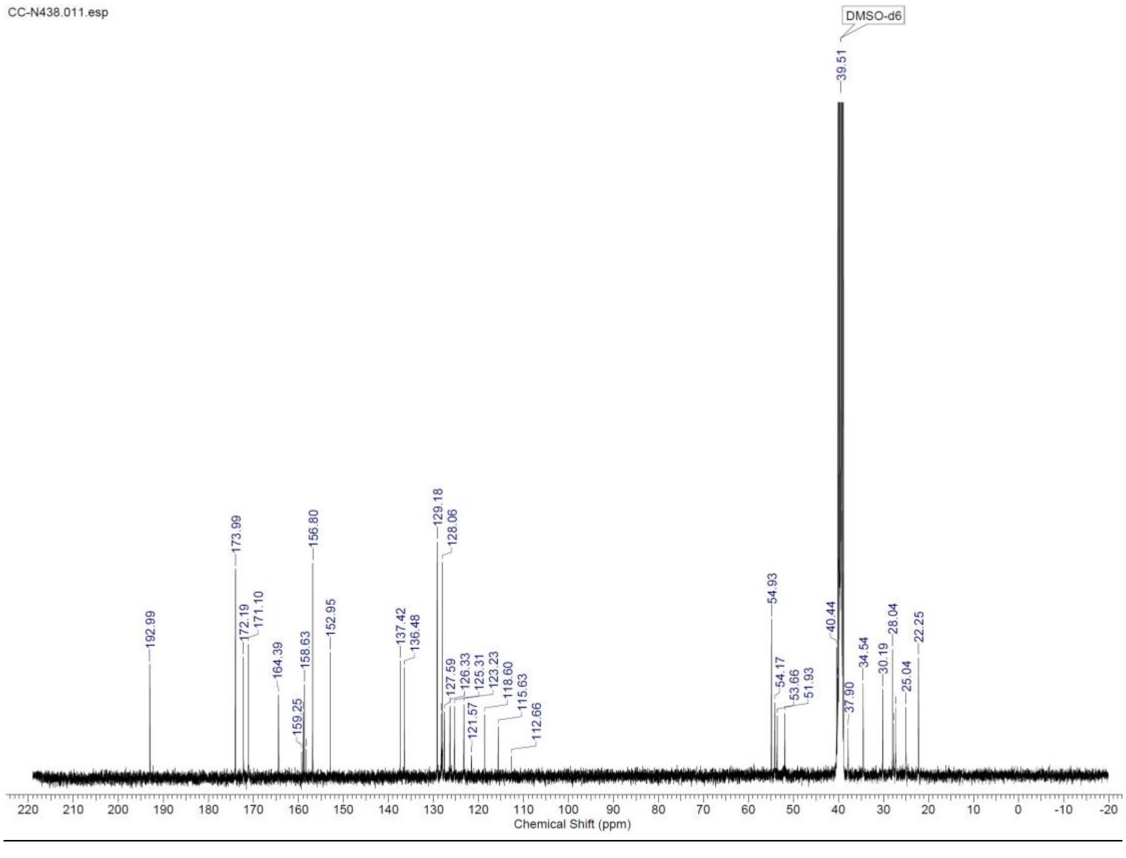

Supplementary Figure 14. ^{1}H-NMR and ^{13}C-NMR of N-0438.

Synthesis of N-1296

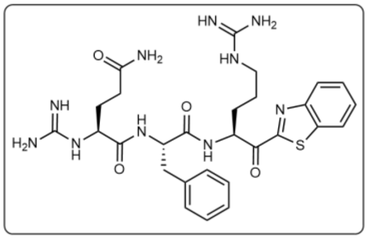

A solution of *N*,*N'*-di-Boc-1*H*-pyrazole-1-carboxamidine (714 mg, 2.30 mmol, 1 eqs.) in DMF (10 mL) was added to the resin containing dipeptide **2**, followed by addition of DMAP (28 mg, 0.23 mmol, 0.1 eq.). The resin was shaken for 3 h, filtrated, washed with 3 x DMF, 1 x *i*-PrOH, 3 x DCM, 1 x Et_2O, then dried (conversion: 74 % by UPLC). Another similar treatment of the resin with *N*,*N'*-di-Boc-1*H*-pyrazole-1-carboxamidine (357 mg, 1.15 mmol, 1 eq.) in DMF (10 mL) and DMAP (14 mg, 0.12 mmol, 0.1 eq.) was made and the resin was shaken for 17 h, filtrated, washed with 3 x DMF, 1 x *i*-PrOH, 3 x DCM, 1 x Et_2O, then dried (conversion: 86 % by UPLC). To the derivatized resin was added a solution of 20 % HFIP in DCM (10 mL) and the mixture was shaken for 15 min. After removal of the solution, the resin was washed with 3 x 10 mL DCM and the solvent was evaporated. The crude material was purified by flash column chromatography on silica gel to give protected dipeptide **24** (418 mg, 47 % global yield based on resin loading).

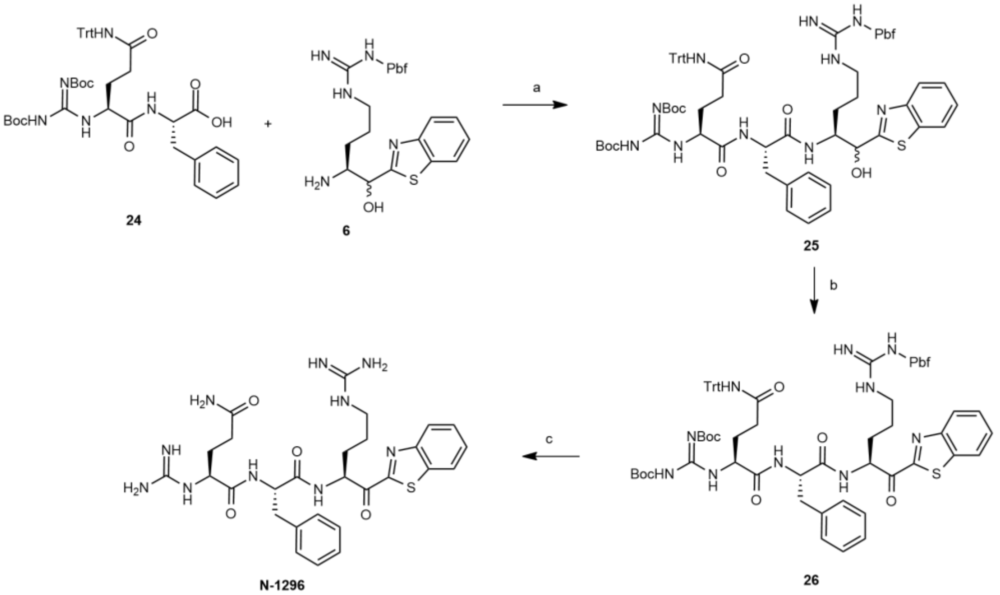

Supplementary Scheme 10. Solution synthesis of N-1296. Reagents and conditions: (a) HATU, DIPEA, DMF, 74% (b) DMP, DCM, 86%. (c) TFA/H_2O (95:5).

A solution of dipeptide **24** (389 mg, 0.50 mmol, 1 eq. (??)) and HATU (209 mg, 0.55 mmol, 1.1 eq.) in DMF (7.5 mL) was cooled to 0 °C. Warhead **6** (320 mg, 0.55 mmol, 1.1 eq.), and DIPEA (261 mL, 1.50 mmol, 3 eqs.) were added and the mixture was stirred at room temperature for 20 min. The protected tripeptide was precipitated in ice, filtrated, and washed with cold water twice. The filtrate was dissolved in DCM and washed with water. The organic phase was dried over $MgSO_4$ and concentrated. The alcohol intermediate (619 mg) was directly used for the next step without purification.

To a solution of the alcohol intermediate **25** (610 mg, 0.47 mmol, 1 eq.) in CH_2Cl_2 (7.5 mL) was added Dess-Martin periodinane (302 mg, 0.71 mmol, 1.5 eq.) The reaction mixture was stirred at room temperature for 20 min. The solution was diluted with DCM, washed with 10 % aq. $Na_2S_2O_3$ and the organic phase was concentrated. The residue was dissolved in AcOEt, washed with sat. aq. $NaHCO_3$, and brine. The organic phase was dried over $MgSO_4$ and concentrated to give the crude material. After purification by flash column chromatography on silica gel, the protected tripeptide **26** (335 mg, 51 % for 2 steps) was obtained.

Protected tripeptide **26** (261 mg, 0.20 mmol) was dissolved in a 95:5 mixture of TFA/H_2O (2.5 mL) and was stirred at room temperature for 1 h. The TFA/H_2O solution was added dropwise to 35 ml of cold Et_2O (–18 °C) in a centrifugation tube and then centrifuged at 4000 rpm for 20 min. The supernatant was removed and the precipitate was dissolved in water, washed with ether and lyophilized. The residue was purified reverse phase prep-HPLC MS (C18) using an ACN/water gradient (0.1% TFA) from 10 to 30% of ACN to give **N-1296** (71 mg, 58%). UPLC-MS retention time: 0.93 min. Purity: >95%.

Am-QFR-Kbt (N-1296):

^{1}H-NMR (400 MHz, d_6-DMSO): δ 9.40 p.p.m. (d, J = 7.9 Hz, 1H), 8.89 (d, J = 6.1 Hz, 1H), 8.79 (br. s, 1H), 8.27 (td, J = 7.8, 1.7 Hz, 2H), 7.72-7.62 (m, 2H), 7.35, 7.30-7.18 (m, 5H), 7.13 (t, J = 7.2 Hz, 1H), 6.79 (s, 1H), 5.50-5.42 (m, 1H), 4.42-4.31 (m, 1H), 4.30-4.21 (m, 1H), 3.18-2.89 (m, 4H), 2.18-2.07 (m, 1H), 2.05-1.62 (m, 6H); ^{13}C-NMR (125 MHz, d_6-DMSO): δ 192.8, 173.9, 173.7, 172.31, 171.9, 170.1, 169.9, 164.6, 164.5, 157.1, 156.8, 156.6, 153.1, 138.0, 137.9, 136.5,

129.4, 128.4, 128.1, 127.7, 126.3, 125.4, 123.3, 55.6, 54.9, 54.5, 31.1, 28.4, 27.4, 24.9; HRMS (*m/z)*: $[M+H]^+$ calcd for $C_{28}H_{36}N_{10}O_4S$, 609.2714; found, 609.2781

Supplementary Table 5. Accurate mass measurement for the compound N-1296

Compound	N-1296<br>Abundant Ion	N-1296<br>Secondary Ion
Structure	$C_{28}H_{36}N_{10}O_4S$	$C_{28}H_{36}N_{10}O_4S$
Analysis	LC-Qtof	LC-Qtof
Electrospray	ESI +	ESI +
Charge	2; $[M+2H]^{2+}$	1; $[M+H]^+$
m/z theorical	305.1393	609.2714
m/z measured	305.1446	609.2781
Δm	0.0053	0.0067
Dissolution solvent	H_2O/MeCN 80/20 +0.1% formic acid (v+v)	

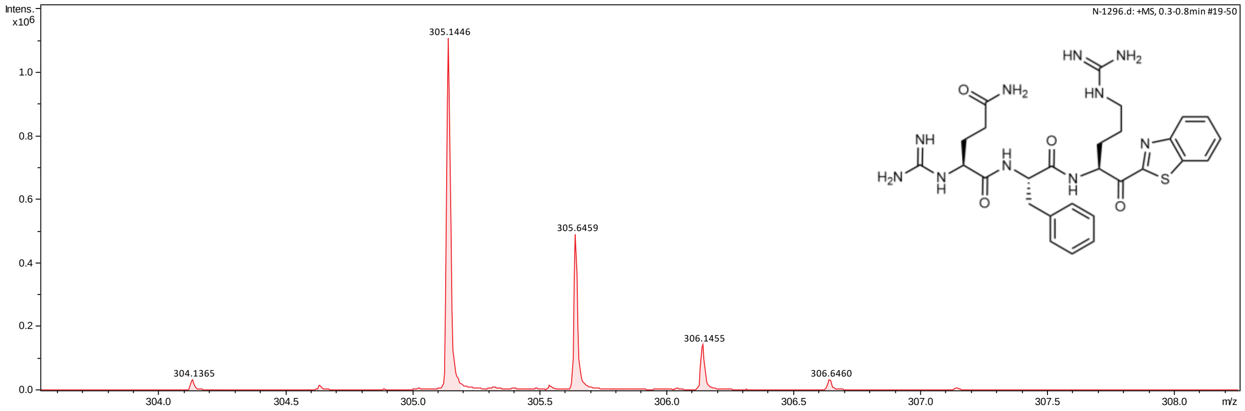

Supplementary Figure 15. Isotopic profile for the most abundant ion (double charged) N-1296, $[M+2H]^{2+}$ detected with high-resolution mass spectrometry (Qtof).

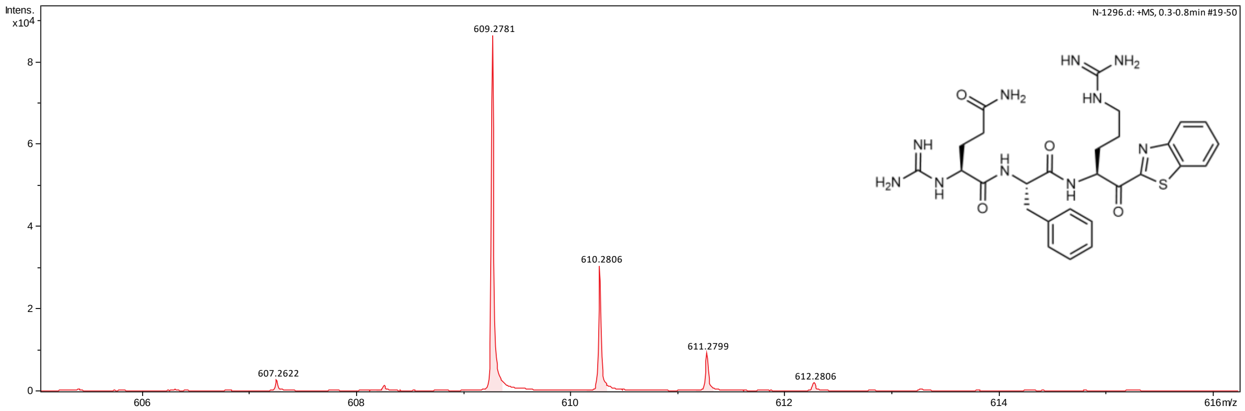

Supplementary Figure 16. Isotopic profile for the secondary ion N-1296, $[M+H]^+$ detected with high-resolution mass spectrometry (Qtof).

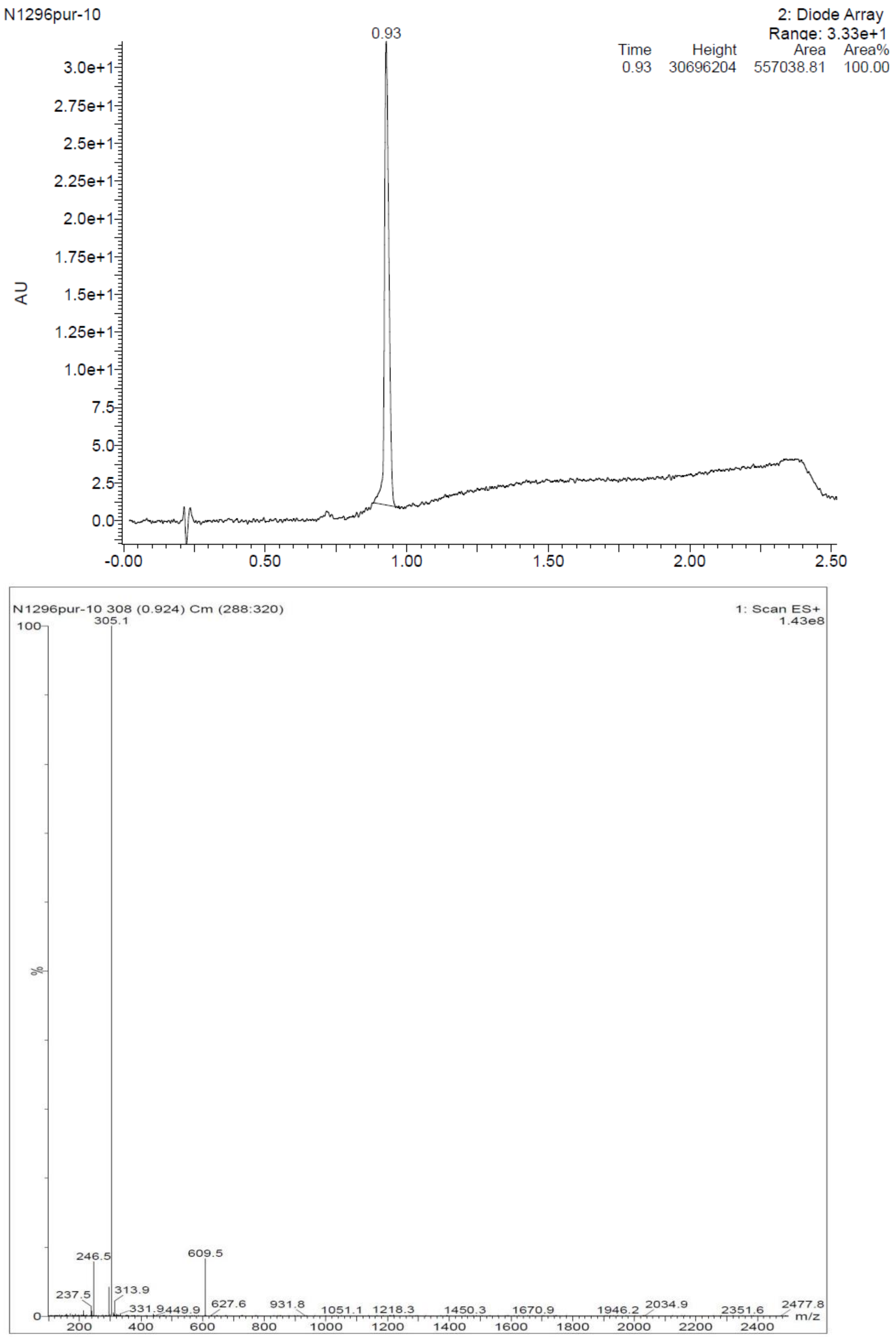

Supplementary Figure 17. UPLC chromatogram and MS of N-1296.

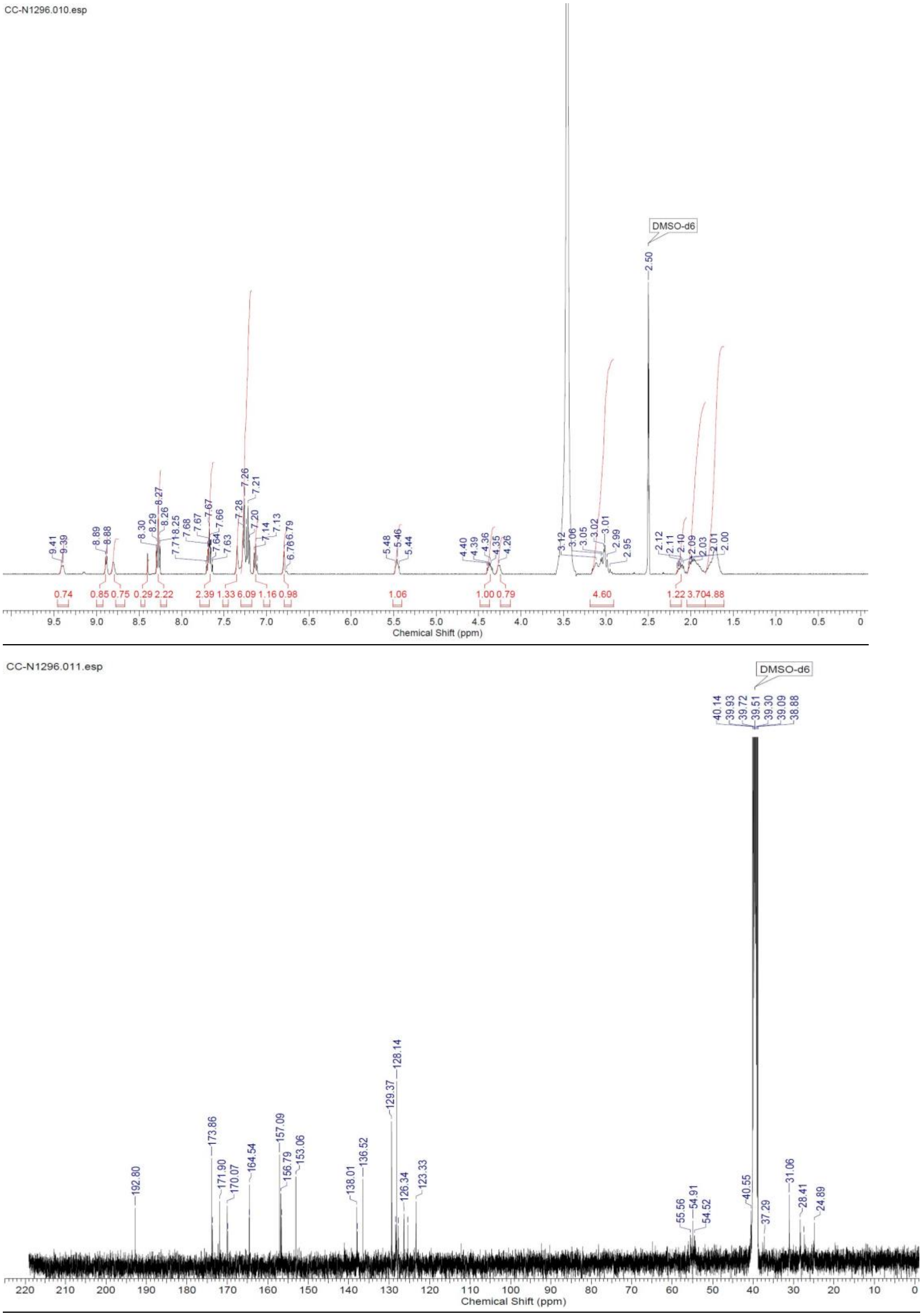

Supplementary Figure 18. ^{1}H-NMR and ^{13}C-NMR of N-1296.

Synthesis of N-0100

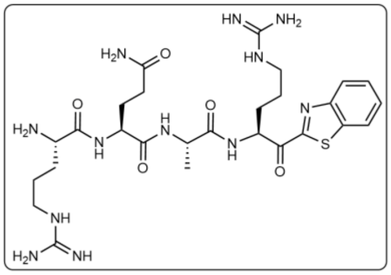

N-0100 was synthesized according to the procedure described for **N-0130** using commercially available Fmoc-Arg(Pbf)-OH instead of **3** and Fmoc-Ala-OH instead of Fmoc-Phe-OH (**Supplementary Scheme 1**).

Compounds N-0100 was published and characterized in *ACS Med. Chem. Lett.* 2012, 3, 7, 530–534.

Synthesis of N-0676

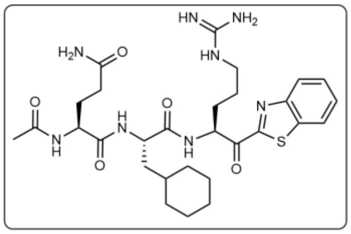

N-0676 was synthesized according to the procedure described for **N-0386** using commercially available Fmoc-Cha-OH instead of Fmoc-Phe-OH. (**Supplementary Scheme 3**). UPLC-MS retention time: 1.29 min. Purity: >95%.

Ac-QChaR-Kbt (N-0676):

^{1}H-NMR (400 MHz, d_6-DMSO) δ 8.66 p.p.m (d, J = 6.2 Hz, 1H), 8.37 (d, J = 7.8 Hz, 1H), 8.30 – 8.22 (m, 3H), 8.16 (s, 1H), 7.71 – 7.61 (m, 2H), 7.39 (s, 1H), 7.26 (s, 3H), 6.74 (s, 1H), 5.45 – 5.37 (m, 1H), 4.34 – 4.25 (m, J = 14.4, 8.9 Hz, 1H), 4.25 – 4.16 (m, J = 8.5, 5.6 Hz, 1H), 3.15 – 3.05 (m, J = 5.3 Hz, 2H), 2.16 – 2.04 (m, J = 13.7, 6.7 Hz, 2H), 2.00 – 1.88 (m, 2H), 1.84 (s, 4H),

1.82 – 1.68 (m, 2H), 1.67 – 1.39 (m, 8H), 1.28 – 1.16 (m, *J* = 7.5 Hz, 1H), 1.14 – 0.95 (m, 3H), 0.86 – 0.67 (m, *J* = 29.2, 11.1 Hz, 2H). ^{13}C-NMR (101 MHz, *d*$_6$-DMSO) δ 192.81, 174.11, 172.74, 171.49, 169.47, 164.57, 156.81, 152.94, 136.38, 128.19, 127.54, 125.26, 123.21, 54.31, 52.72, 50.43, 40.37, 33.36, 33.06, 31.91, 31.78, 28.10, 27.28, 26.05, 25.77, 25.55, 24.81, 22.55; HRMS (*m/z*): $[M+H]^+$ calcd for $C_{29}H_{42}N_8O_5S$, 615.3071; found, 615.3155

Supplementary Table 6. Accurate mass measurement for the compound N-0676

Compound	N-0676
Structure	$C_{29}H_{42}N_8O_5S$
Analysis	LC-Qtof
Electrospray	ESI +
Charge	1; $[M+H]^+$
m/z theorical	615.3071
m/z measured	615.3155
Δm	0.0084
Dissolution solvent	H_2O/MeCN 80/20 +0.1% formic acid (v+v)

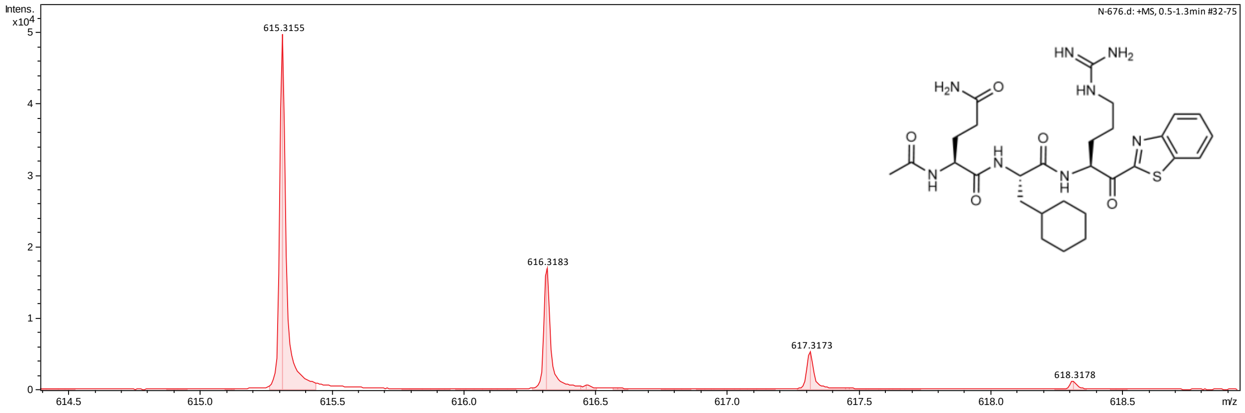

Supplementary Figure 19. Isotopic profile for the compound N-0676, $[M+H]^+$ detected with high-resolution mass spectrometry (Qtof).

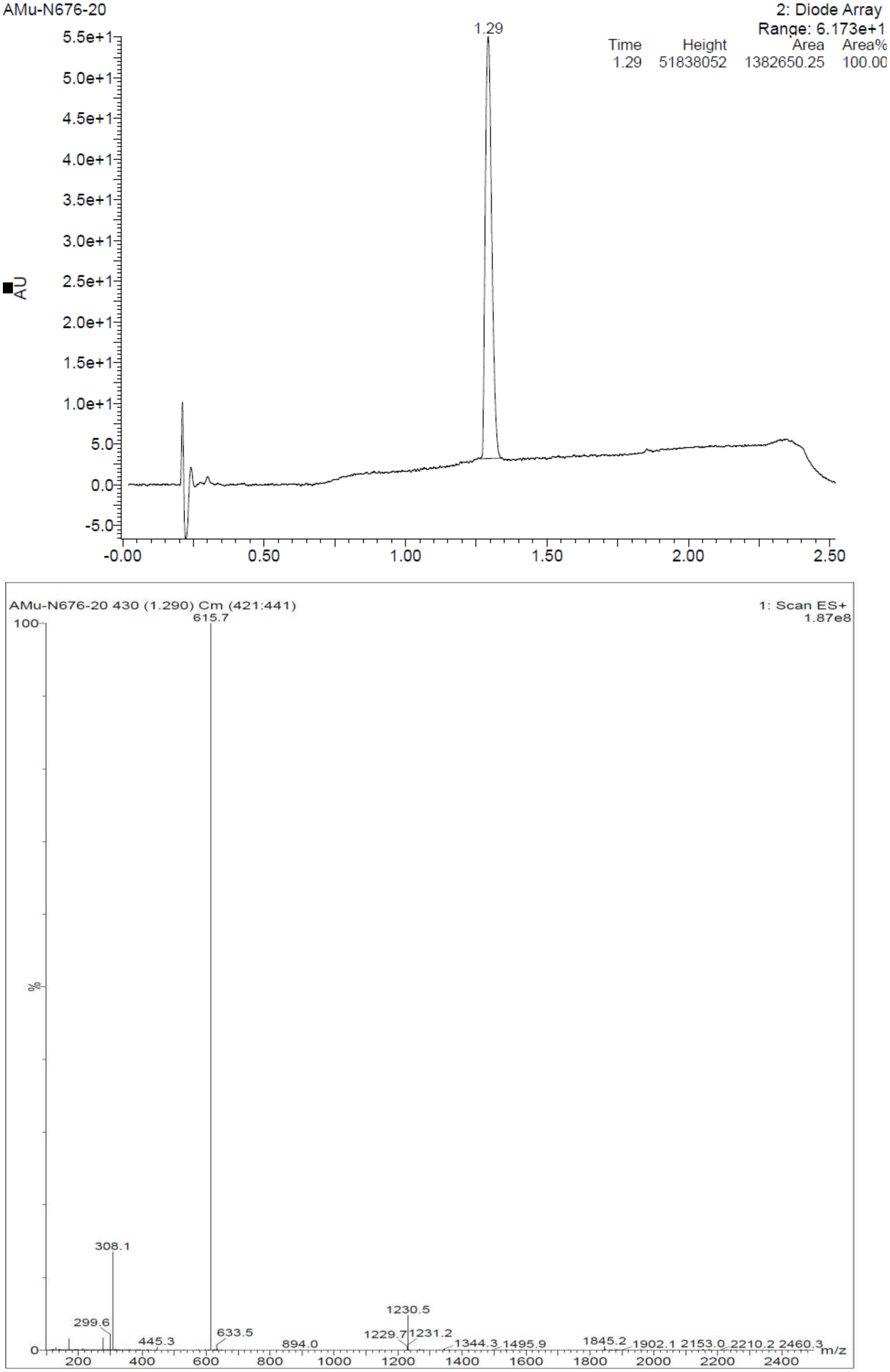

Supplementary Figure 20. UPLC chromatogram and MS of N-0676.

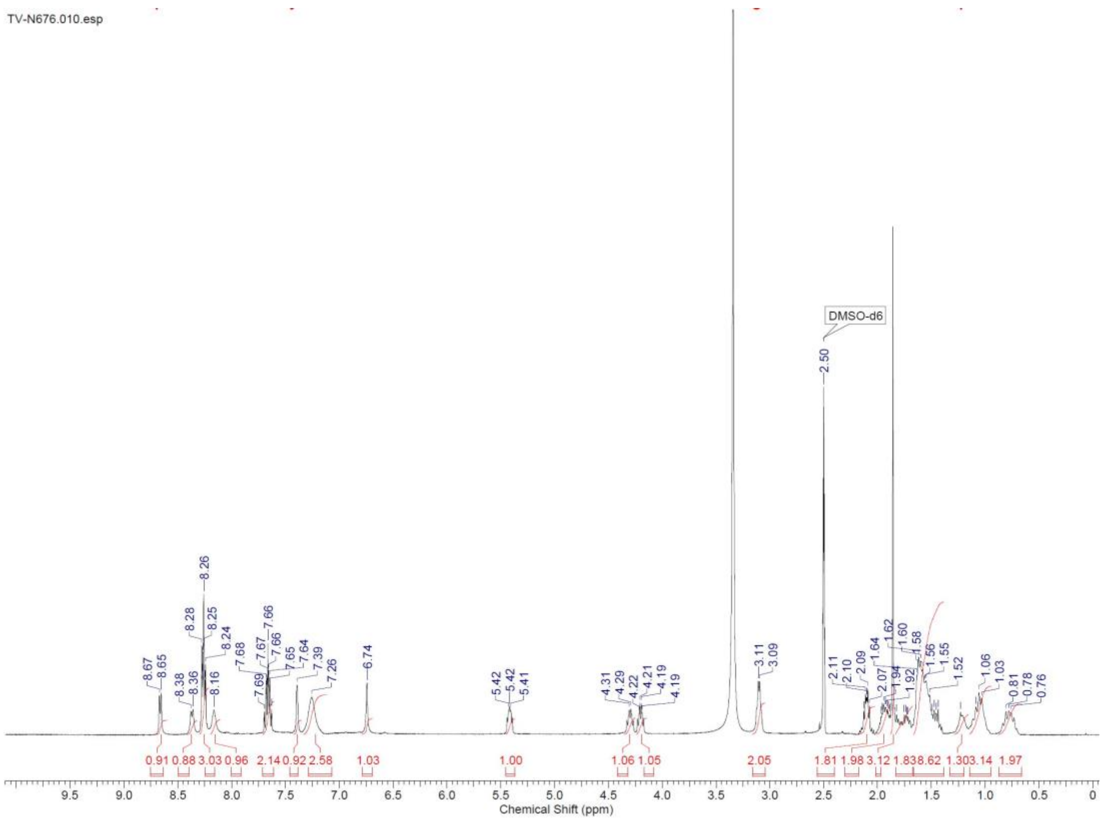

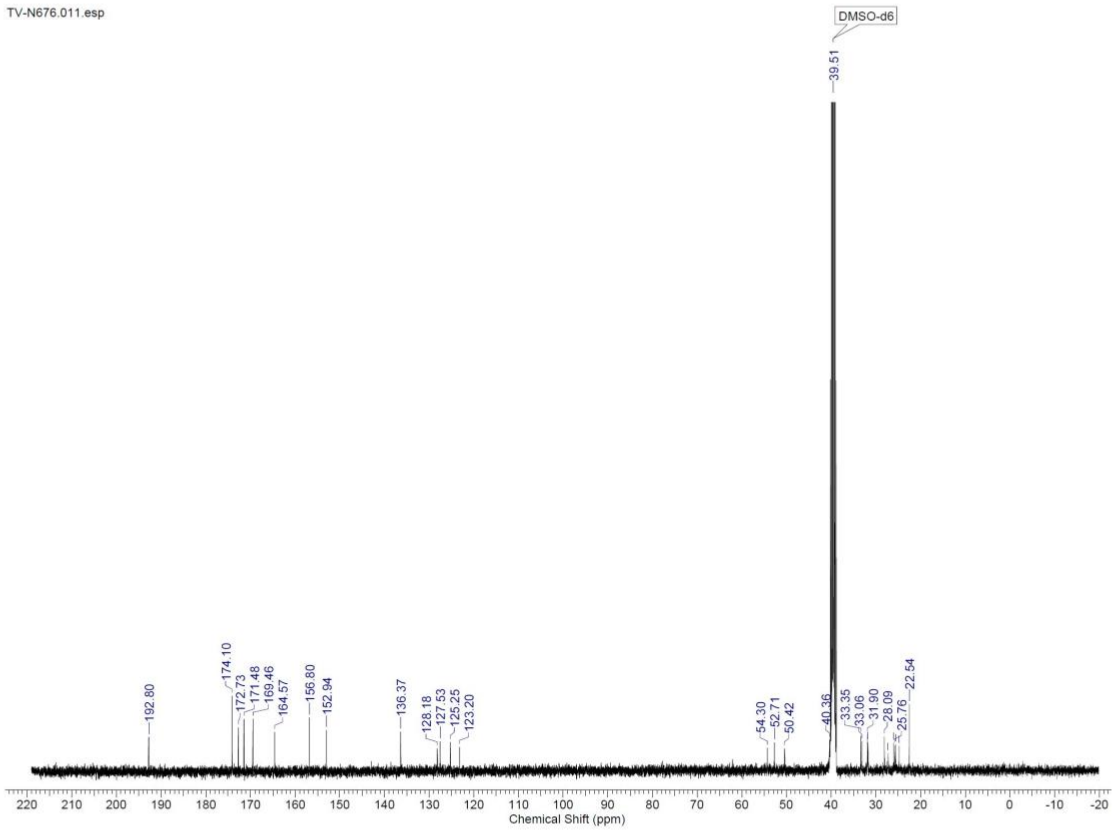

Supplementary Figure 21. ^{1}H-NMR and ^{13}C-NMR of N-0676.

Synthesis of N-0678

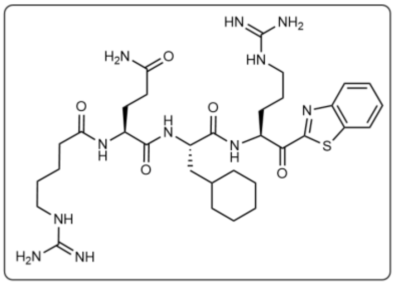

N-0678 was synthesized according to the procedure described for **N-0130** using commercially available Fmoc-Cha-OH instead of Fmoc-Phe-OH (**Supplementary Scheme 1**). UPLC-MS retention time: 1.03 min. Purity: >95%.

(H)RQChaR-Kbt (N-0678):

^{1}H-NMR (400 MHz, *d$_6$*-DMSO): δ 8.63 p.p.m. (d, *J* = 6.8 Hz, 1H), 8.30-8.22 (m, 2H), 8.02 (dd, *J* = 16.0, 7.8 Hz, 1H), 7.97-7.84 (m, 2H), 7.78 (br. s, 1H), 7.72-7.62 (m, 2H), 7.29 (d, *J* = 11.6 Hz, 2H), 6.8 (br. s, 1H), 5.47-5.36 (m, 1H), 4.45-4.34 (m, 1H), 4.27-4.16 (m, 1H), 3.17-3.01 (m, 4H), 2.22-2.02 (m, 4H), 2.02-1.91 (m, 1H), 1.89-1.78 (m, 1H), 1.78-1.30 (m, 18H), 1.22-1.16 (m, 1H), 1.16-0.94 (m, 3H), 0.90-0.72 (m, 2H). ^{13}C-NMR (125 MHz, *d$_6$*-DMSO): δ 193.1, 193.0, 174.0, 173.9, 172.5, 172.4, 172.3, 172.1, 171.4, 171.3, 167.3, 164.5, 164.5, 158.9, 158.6, 157.0, 156.9, 152.9, 136.4, 136.4, 128.2, 127.6, 125.3, 123.2, 118.6, 115.7, 54.4, 54.3, 52.4, 52.2, 49.9, 40.4, 40.3, 34.6, 33.4, 33.3, 33.1, 31.9, 31.6, 28.1, 27.9, 27.7, 27.5, 27.4, 26.1, 25.8, 25.5, 25.2, 25.0, 22.3; HRMS *(m/z)*: $[M+2H]^{2+}$ calcd for $C_{33}H_{51}N_{11}O_5S$, 357.6970; found, 357.7030

Supplementary Table 7. Accurate mass measurement for the compound N-0678.

Compound	N-0678
Structure	$C_{33}H_{51}N_{11}O_5S$
Analysis	LC-Qtof
Electrospray	ESI +
Charge	2; $[M+2H]^{2+}$
m/z theorical	357.6970
m/z measured	357.7030
Δm	0.0060
Dissolution solvent	H_2O/MeCN 80/20 +0.1% formic acid (v+v)

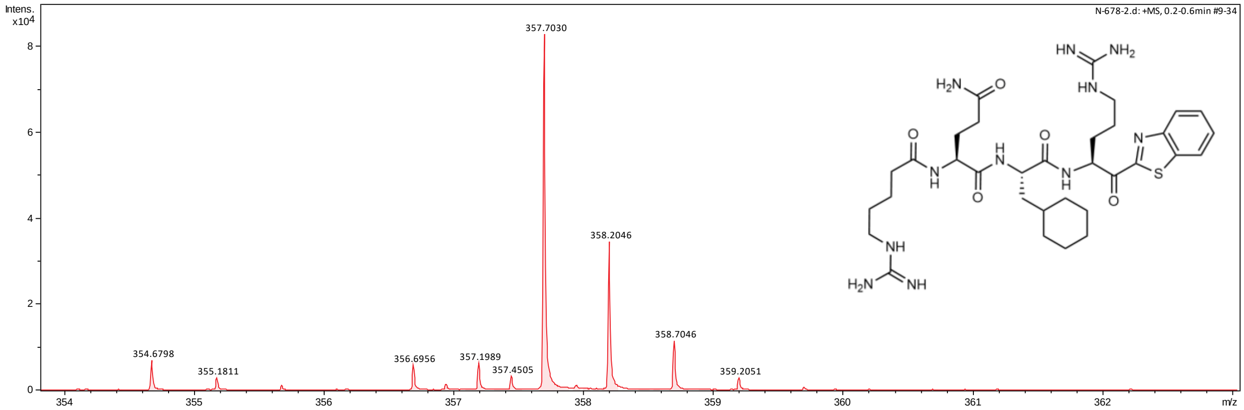

Supplementary Figure 22. Isotopic profile for the compound N-0678, $[M+2H]^{2+}$ detected with high-resolution mass spectrometry (Qtof).

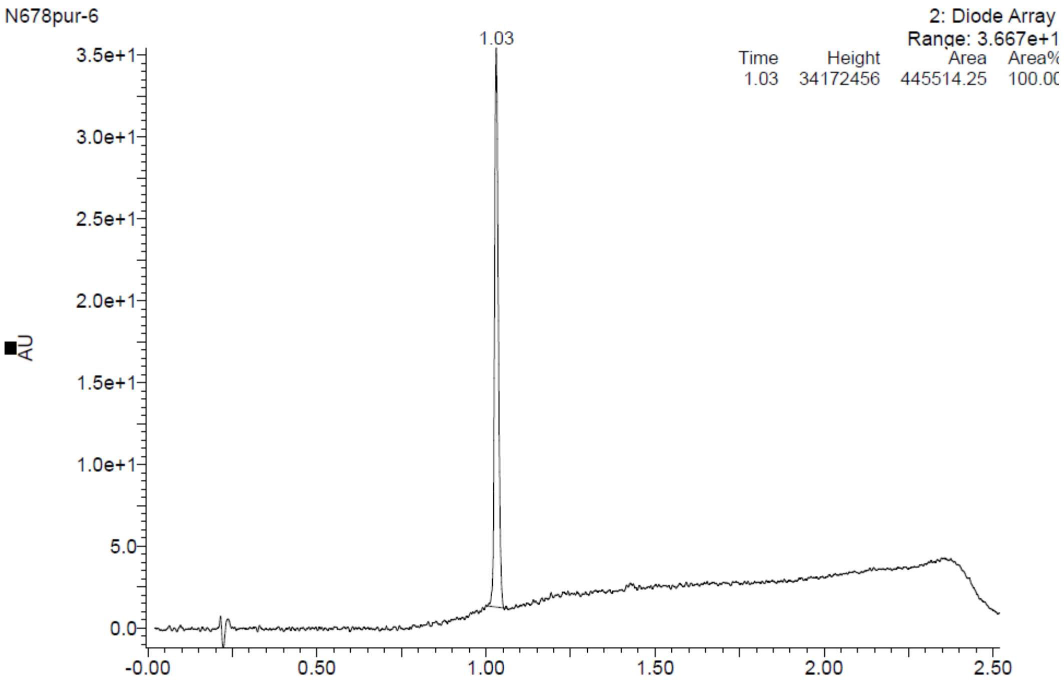

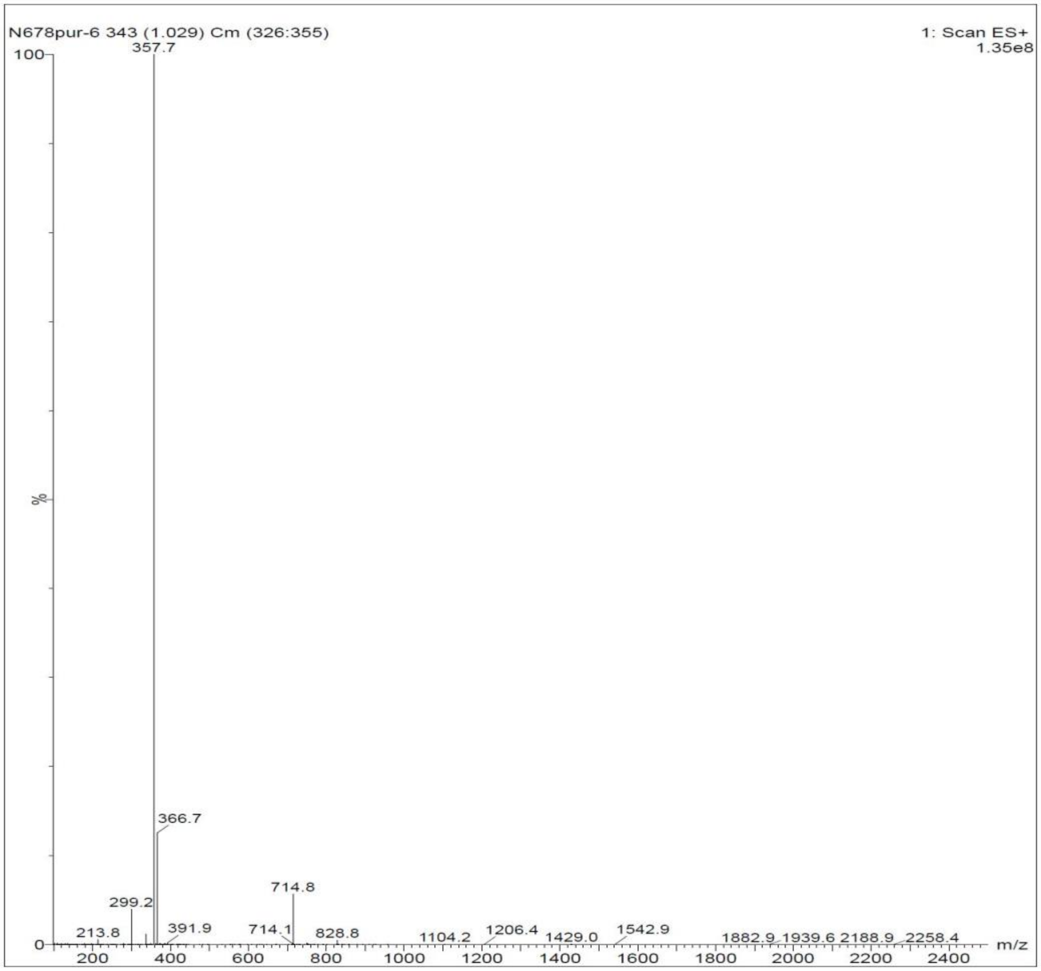

Supplementary Figure 23. UPLC chromatogram and MS of N-0678.

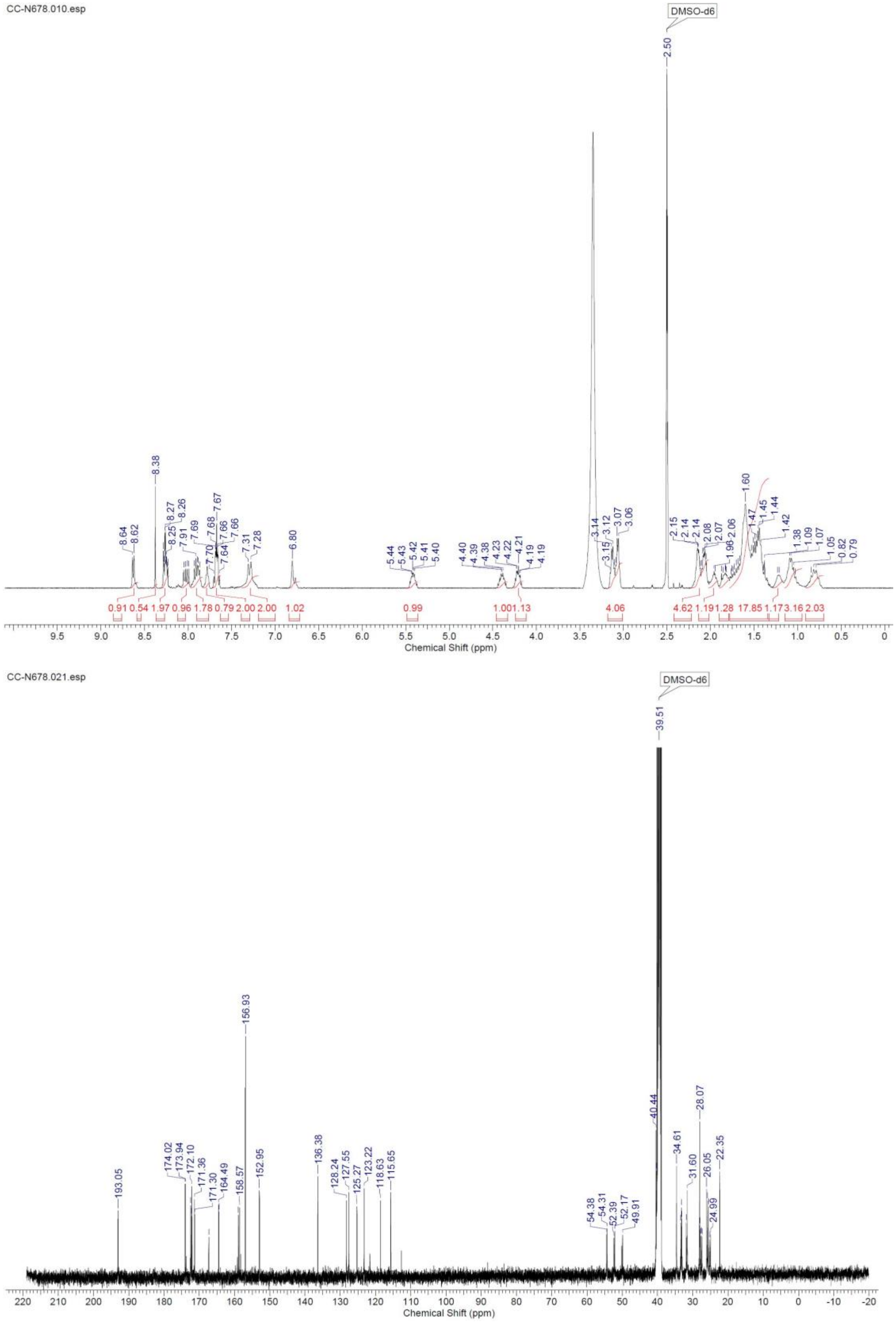

Supplementary Figure 24. ^{1}H-NMR and ^{13}C-NMR of N-0678.

Synthesis of N-0385(OH)

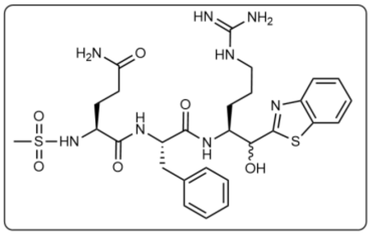

N-0385(OH) was synthesized according to the procedure described for **N-0385** without the oxydation step and obtain as a racemic mixture. UPLC-MS retention time: 0.94 min. Purity: >95%.

Ms-QFR-Kbt(OH) (N-0385(OH):

^{1}H-NMR (400 MHz, *d6*-DMSO): δ 8.27-8.20 p.p.m. (m, 2H), 8.13-8.07 (m, 1H), 7.96- 7.87 (m, 1H), 7.54- 7.38 (m, 3H), 7.37 (s, 1H), 7.33-7.28 (m, 1 H), 7.26-7.13 (m, 6H), 6.85 (s, 1H), 6.81-6.76 (m, 1H), 4.90-4.84 (m, 1H), 4.64-4.56 (m, 1H), 4.33-4.22 (m, 1H), 3.70-3.60 (m, 1H), 3.08-2.93 (m, 3H), 2.59-2.53 (m, 1H), 2.41 (s, 3H), 2.21-2.09 (m, 1H), 2.07-1.96 (m, 1H), 1.79-1.66 (m, 1H), 1.64-1.43 (m, 4H), 1.41-1.29 (m, 1H); ^{13}C-NMR (125 MHz, *d6*-DMSO): δ 176.3, 173.9, 171.0, 170.9, 156.6, 153.1, 153,1, 137.7, 134.4, 129.0, 128.0, 126.3, 126.0, 124.8, 122.5, 122.3, 73.0, 56.0, 53.6, 53.2, 40.6, 37.6, 31.3, 28.9, 25.9, 25.3; HRMS (*m/z)*: $[M+H]^+$ calcd for $C_{28}H_{38}N_8O_6S$, 647.2428; found, 647.2506

Supplementary Table 8. Accurate mass measurement for the compound N-0385-OH

Compound	N-0385(OH)
Structure	$C_{28}H_{38}N_8O_6S_2$
Analysis	LC-Qtof
Electrospray	ESI +
Charge	1; $[M+H]^+$
m/z theorical	647.2428
m/z measured	647.2506
Δm	0.0078
Dissolution solvent	H_2O/MeCN 80/20 +0.1% formic acid (v+v)

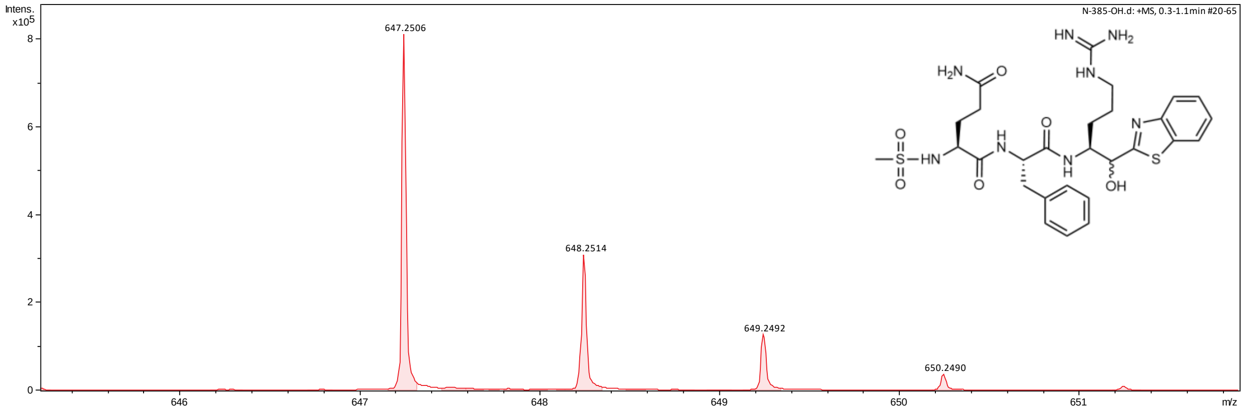

Supplementary Figure 25. Isotopic profile for the compound N-0385(OH), [M+H] + detected with high-resolution mass spectrometry (Qtof)

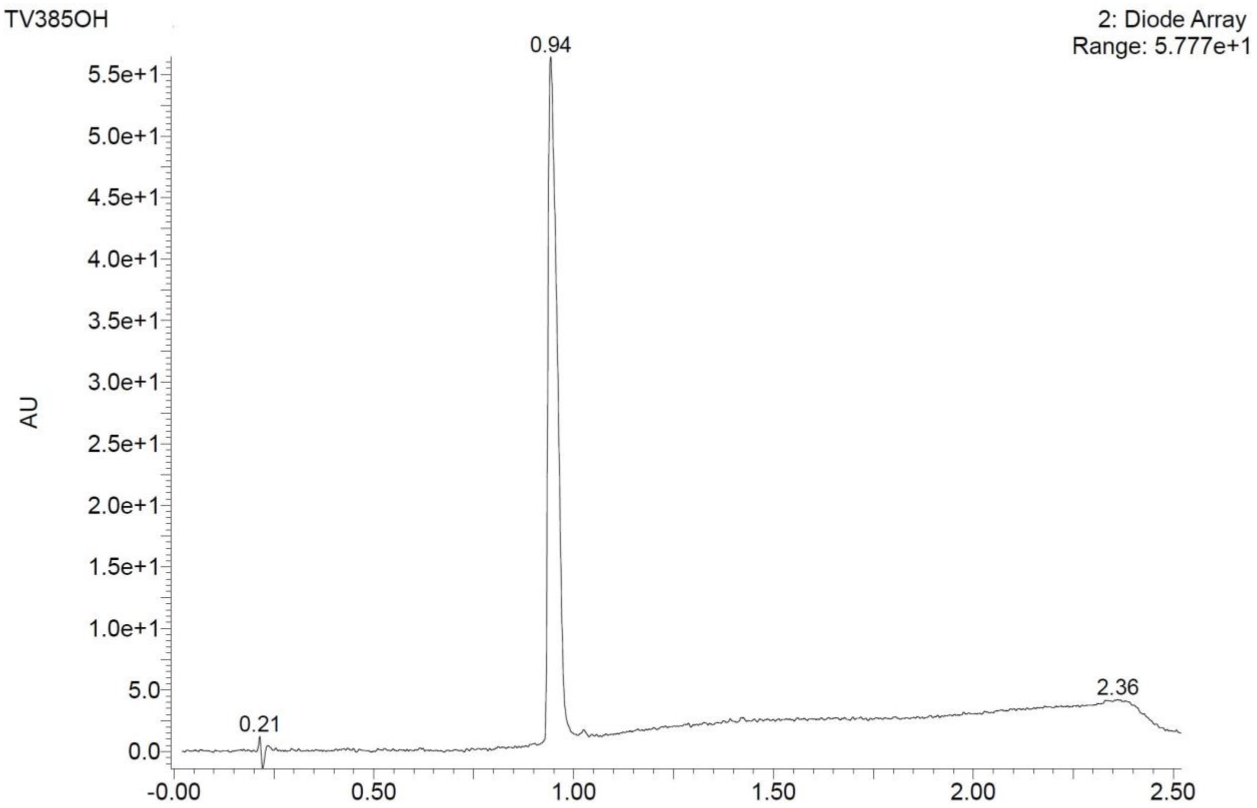

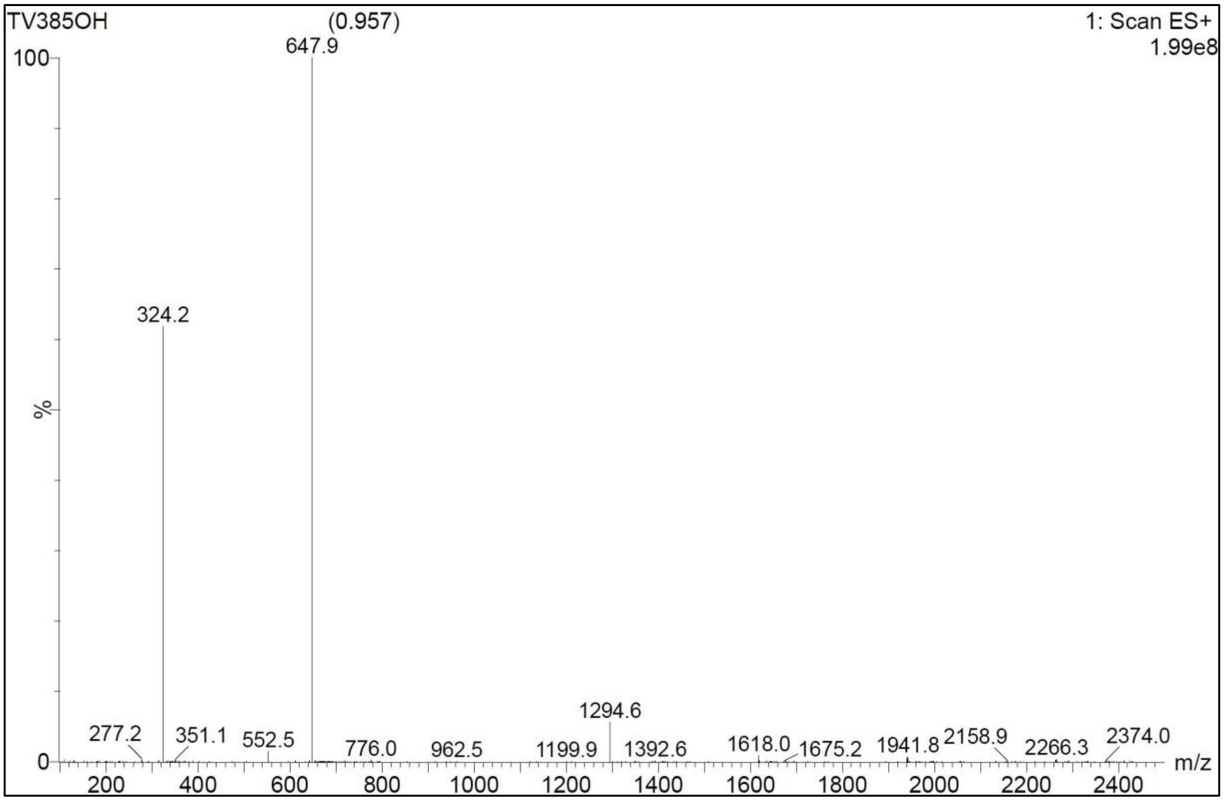

Supplementary Figure 26. UPLC chromatogram and MS of N-0385(OH) (mixture of diastereoisomers).

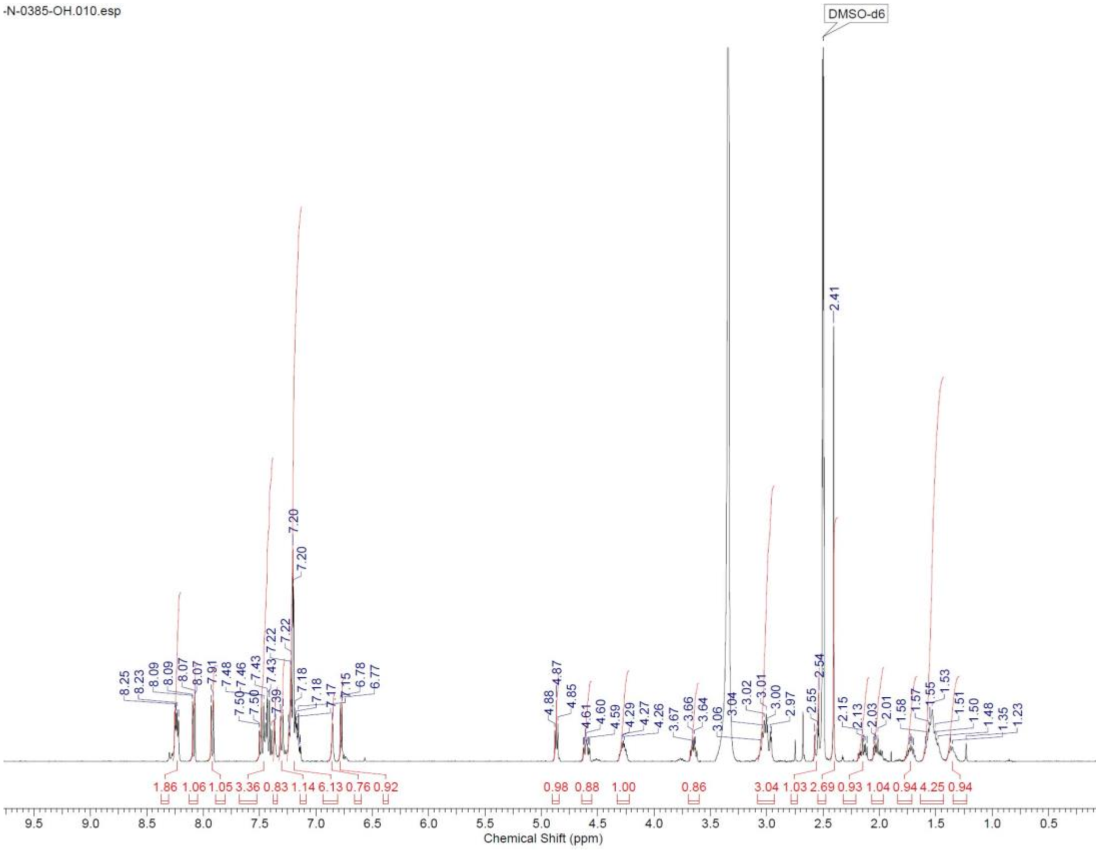

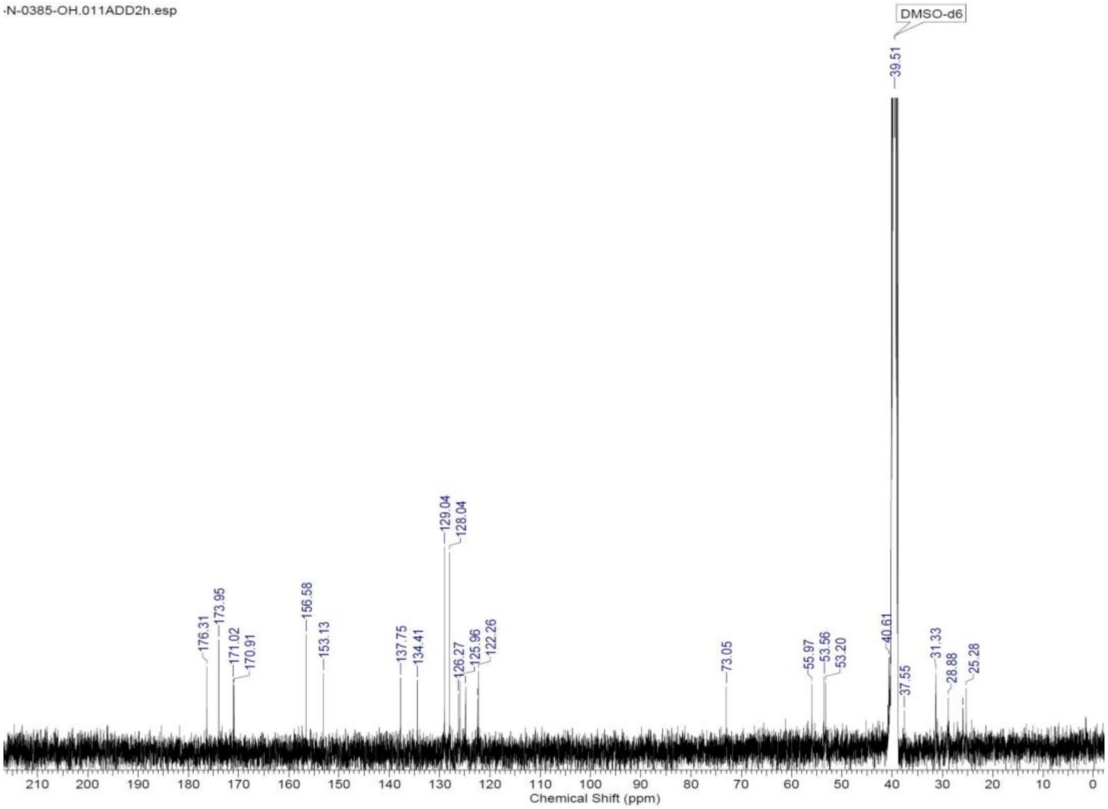

Supplementary Figure 27. ^{1}H-NMR and ^{13}C-NMR of N-0385(OH) (mixture of diastereoisomers)
