# Supplementary material for: A﻿ TMPRSS2 inhibitor acts as a pan-SARS-CoV-2 prophylactic and therapeutic
Source: Nature. 2022 Mar 28;605(7909):340–8. doi: 10.1038/s41586-022-04661-w (PMC9095466; doi:10.1038/s41586-022-04661-w)
Supplement: Supplementary file 2 — Reporting Summary [file 41586_2022_4661_MOESM2_ESM.pdf]

nature research

Corresponding author(s): Dr. François Jean, Dr. Richard Leduc, Dr. Hector C. Aguilar

Last updated by author(s): January 18, 2022

Reporting Summary

Nature Research wishes to improve the reproducibility of the work that we publish. This form provides structure for consistency and transparency in reporting. For further information on Nature Research policies, see our Editorial Policies and the Editorial Policy Checklist.

Statistics

For all statistical analyses, confirm that the following items are present in the figure legend, table legend, main text, or Methods section.

n/a	Confirmed	
☐	☒	The exact sample size (n) for each experimental group/condition, given as a discrete number and unit of measurement
☐	☒	A statement on whether measurements were taken from distinct samples or whether the same sample was measured repeatedly
☐	☒	The statistical test(s) used AND whether they are one- or two-sided<br>*Only common tests should be described solely by name; describe more complex techniques in the Methods section.*
☒	☐	A description of all covariates tested
☐	☒	A description of any assumptions or corrections, such as tests of normality and adjustment for multiple comparisons
☐	☒	A full description of the statistical parameters including central tendency (e.g. means) or other basic estimates (e.g. regression coefficient) AND variation (e.g. standard deviation) or associated estimates of uncertainty (e.g. confidence intervals)
☐	☒	For null hypothesis testing, the test statistic (e.g. F, t, r) with confidence intervals, effect sizes, degrees of freedom and P value noted<br>*Give P values as exact values whenever suitable.*
☒	☐	For Bayesian analysis, information on the choice of priors and Markov chain Monte Carlo settings
☒	☐	For hierarchical and complex designs, identification of the appropriate level for tests and full reporting of outcomes
☒	☐	Estimates of effect sizes (e.g. Cohen's d, Pearson's r), indicating how they were calculated

Our web collection on statistics for biologists contains articles on many of the points above.

Software and code

Policy information about availability of computer code

Data collection	No software was used to collect data in this study
Data analysis	Molecular Operating Environment (MOE2019.01.02) from the Chemical Computing Group used to generate the TMPRSS2 model with N-0385; (HCS Studio Cell Analysis Software, version 4.0) was used to quantify viral markers of SARS-CoV-2 infection; The GraphPad Prism 9™ (GraphPad Software, Inc.) was used for statistical analysis; Digital image analysis for mouse/nucleocapsid staining was performed using QuPath software version 0.2.3; Leica Application Suite X (LAS X) software was used for acquiring confocal images. Merging of different channels from confocal imaging and the addition of the scale bar were performed using ImageJ/FIJI.

For manuscripts utilizing custom algorithms or software that are central to the research but not yet described in published literature, software must be made available to editors and reviewers. We strongly encourage code deposition in a community repository (e.g. GitHub). See the Nature Research guidelines for submitting code & software for further information.

Data

Policy information about availability of data

All manuscripts must include a data availability statement. This statement should provide the following information, where applicable:

- Accession codes, unique identifiers, or web links for publicly available datasets
- A list of figures that have associated raw data
- A description of any restrictions on data availability

All data generated or analyzed during this study are included in this published article (and its supplementary information files).

Field-specific reporting

Please select the one below that is the best fit for your research. If you are not sure, read the appropriate sections before making your selection.

☒ Life sciences ☐ Behavioural & social sciences ☐ Ecological, evolutionary & environmental sciences

For a reference copy of the document with all sections, see nature.com/documents/nr-reporting-summary-flat.pdf

Life sciences study design

All studies must disclose on these points even when the disclosure is negative.

Sample size	Ten mice were used per group based on the following power analysis statistical power calculations: We sought a sample size that was large enough to detect significant differences in the EC50 averages (+/- standard deviations) between groups. We sought help from the Cornell Statistical Consulting Unit to perform our power analysis. For this, we used our pilot data and a 2-group, 2 sided analysis, setting our power (1-) to 0.9, to have 90% power to detect a statistical difference, and an error rate of 5%, to achieve a significance level of corresponding to a 5% error. These calculations resulted in an N number of animals per groups of seven. However, 10 animals were used to ensure statistical significance in the case of unexpected animal deaths.<br><br>No sample size calculations were performed for in vitro and cell culture experiments. Sample sizes were based on standards in the field, typically 3 independent biological experiments with each replicate assayed in technical triplicate or greater. Peptidomimetic screening (Fig 2a) against SARS-CoV-2 in Calu-3 was performed only twice (two independent experiments with 5 technical replicates each) to identify the lead compound for detailed downstream analysis. Real-time qPCR was only performed once to qualitatively verify the expression of ACE2 and TMPRSS2 in Calu-3 and colonoids. Due to limited sample availability, the control compound N-0385(OH) was only evaluated against SARS-CoV-2 twice in colonoids and the results were consistent in both experiments. Note that the active compounds N-0385 was tested independently three times in colonoids against SARS-CoV-2.
Data exclusions	1 male animal was excluded from the N-0385(OH) control group that died of unknown causes (day -1-6 treatment), that did not affect statistical significance when excluded or included. GraphPad Prism was used to identify and eliminate outliers (Q = 1) for inhibitor screening, IC50 determination and protease selectivity (Ki).
Replication	Each experiment using 10 mice per treatment group (5 female and 5 male) was performed once. The number of animals used is sufficient to interpret the results with an error rate <5% using male and female animals precluding the necessity of conducting animal experiments in replicate. Also, each animal is considered an independent biological replicate.
Randomization	Mice were designated into groups randomly to reduce bias due to differences in weight, and animal studies were performed using age matched mice to compare across groups.<br><br>Randomization does not apply to cell-based and in vitro experiments, in which large numbers of cells or reagent aliquots from a given source are partitioned among experimental conditions.
Blinding	Investigators were not blinded to groups during weighing and scoring of animal health due to nature of the infectious agent but histopathology, IHC, and related data analyses were dealt with in a blinded fashion by certified pathologist and clinicians in the team.<br><br>Blinding was also not done for in vitro or cell-based experiments as no subjective rating of data was involved. Quantification was automated using plate readers, qPCR machines, high-content screening platforms.

Reporting for specific materials, systems and methods

We require information from authors about some types of materials, experimental systems and methods used in many studies. Here, indicate whether each material, system or method listed is relevant to your study. If you are not sure if a list item applies to your research, read the appropriate section before selecting a response.

Materials & experimental systems

n/a	Involved in the study	
☐	☒	Antibodies
☐	☒	Eukaryotic cell lines
☒	☐	Palaeontology and archaeology
☐	☒	Animals and other organisms
☒	☐	Human research participants
☒	☐	Clinical data
☐	☒	Dual use research of concern

Methods

n/a	Involved in the study	
☒	☐	ChIP-seq
☒	☐	Flow cytometry
☒	☐	MRI-based neuroimaging

Antibodies

Antibodies used	The SARS-CoV-2 nucleocapsid antibody [HL344] (GTX635679) was provided by Genetex; mouse anti-dsRNA antibody (J2-1904) was purchased from Scicons English and Scientific Consulting; Hoechst 33258 and secondary antibodies goat anti-mouse IgG Alexa Fluor 488 (A11001) and goat anti-rabbit IgG Alexa Fluor 555 (A21428) were obtained from Invitrogen
Validation	The nucleocapsid antibody was validated by the company (Genetex) using validation protocols in line with guidelines described by the International Working Group on Antibody Validation (IWGAV). Detailed validation and testing are available here: https://www.genetex.com/Product/Detail/SARS-CoV-2-COVID-19-nucleocapsid-antibody-HL344/GTX635679#datasheet. The manufacturer also states that "This antibody detects SARS-CoV-2 nucleocapsid protein, but does not cross-react with SARS-CoV or MERS-CoV nucleocapsid proteins based on our internal testing."<br>The dsRNA antibody was validated by SCICONS: https://www.jenabioscience.com/rna-technologies/rna-analysis-detection/dsrna-detection/rnt-sci-10010-anti-dsrna-monoclonal-antibody-j2. The manufacturer states: "Specificity: Anti-dsRNA monoclonal antibody J2 recognises double-stranded RNA (dsRNA) provided that the length of the helix is greater than or equal to 40 bp. dsRNA-recognition is independent of the sequence and nucleotide composition of the antigen. All naturally occurring dsRNAs investigated up to now (40-50 species) as well as poly(I)-poly(C) and poly(A)-poly(U) have been recognised by Anti-dsRNA monoclonal antibody J2 although in some assays its affinity to poly(I)-poly(C) is about 10 times lower than that to other dsRNA antigens."<br>Secondary antibodies and Hoecsht were validated by Invitrogen / Thermo Fisher<br>Both nucleocapsid and dsRNA antibodies were additionally verified for specificity using SARS-CoV-2 infected versus non-infected cells as shown in Extended Data Figure 2 and 4 (see mock vs. SARS-CoV-2 panels) where the antibody signal is not detected in non-infected / mock cells.

Eukaryotic cell lines

Policy information about cell lines

Cell line source(s)	All cell lines (Calu-3 cells (ATCC® HTB-55™) and Vero E6 cells (ATCC® CRL-1586™)) were purchased from ATCC (American Type Culture Collection)
Authentication	None of the cell lines used were authenticated
Mycoplasma contamination	We confirm that all cell lines used tested negative for mycoplasma contamination
Commonly misidentified lines<br>(See ICLAC register)	No commonly misidentified cell lines were used in this study

Animals and other organisms

Policy information about studies involving animals; ARRIVE guidelines recommended for reporting animal research

Laboratory animals	Eight-week-old heterozygous K18 hACE2 c57BL/6J male and female mice (strain: 2B6.Cg-Tg(K18-hACE2)2Prlmn/J) were obtained directly from or bred at Cornell using Jackson Laboratory obtained animals. The rodent housing setting includes a standard 12:12 dark/light cycle (for non-breeding rooms), with a temperature range of 68-72 F (20 to 22 °C), and a relative humidity in the range of 40-60%.
Wild animals	The study did not involve wild animals
Field-collected samples	No field collected samples were used in this study
Ethics oversight	Studies were approved and supervised following protocols set and reviewed under Institutional Animal Care and Use Committee at Cornell University (IACUC mouse protocol # 2017-0108 and BSL3 IBC # MUA-16371-1)

Note that full information on the approval of the study protocol must also be provided in the manuscript.

Dual use research of concern

Policy information about dual use research of concern

Hazards

Could the accidental, deliberate or reckless misuse of agents or technologies generated in the work, or the application of information presented in the manuscript, pose a threat to:

No	Yes	
☐	☒	Public health
☐	☒	National security
☒	☐	Crops and/or livestock
☒	☐	Ecosystems
☒	☐	Any other significant area

Hazards	Use of SARS-CoV-2 and SARS-CoV-2 variants of concern (risk group 3 agent)

For examples of agents subject to oversight, see the United States Government Policy for Institutional Oversight of Life Sciences Dual Use Research of Concern.

Experiments of concern

Does the work involve any of these experiments of concern:

No	Yes	
☒	☐	Demonstrate how to render a vaccine ineffective
☒	☐	Confer resistance to therapeutically useful antibiotics or antiviral agents
☒	☐	Enhance the virulence of a pathogen or render a nonpathogen virulent
☒	☐	Increase transmissibility of a pathogen
☒	☐	Alter the host range of a pathogen
☒	☐	Enable evasion of diagnostic/detection modalities
☒	☐	Enable the weaponization of a biological agent or toxin
☒	☐	Any other potentially harmful combination of experiments and agents

Precautions and benefits

Biosecurity precautions	All infections were carried out in a Biosafety Level 3 (BSL3) facility (UBC FINDER) in accordance with the Public Health Agency of Canada and UBC FINDER regulations (UBC BSL3 Permit # B20-0105 to FJ). Animal studies were performed under approved BSL-3 conditions and approved by the Institutional Animal Care and Use Committee at Cornell University (IACUC mouse protocol # 2017-0108 and BSL3 IBC # MUA-16371-1). All animal experiments were performed under USA CDC and USDA guidelines.
Biosecurity oversight	FINDER is UBC's shared platform for researchers working with Risk Group 3 pathogens. Bio-security oversight is provided by The FINDER Steering Committee members and the UBC Vice-President, Research and Innovation Group. All animal BSL-3 work at Cornell University is reviewed by both the IACUC and IBC committees.
Benefits	Benefit is that this work may mitigate risks to public health by providing new antiviral treatment options for SARS-CoV-2 associated disease (COVID-19)
Communication benefits	There are no risks to communicating this information
